# Phosphate Coatings Enriched with Copper on Titanium Substrate Fabricated Via DC-PEO Process

**DOI:** 10.3390/ma13061295

**Published:** 2020-03-13

**Authors:** Krzysztof Rokosz, Tadeusz Hryniewicz, Wojciech Kacalak, Katarzyna Tandecka, Steinar Raaen, Sofia Gaiaschi, Patrick Chapon, Winfried Malorny, Dalibor Matýsek, Kornel Pietrzak, Łukasz Dudek

**Affiliations:** 1 Department of Engineering and Informatics Systems, Faculty of Mechanical Engineering, Koszalin University of Technology, 75620 Koszalin, Poland; Tadeusz.Hryniewicz@tu.koszalin.pl (T.H.); Wojciech.Kacalak@tu.koszalin.pl (W.K.); Katarzyna.Tandecka@tu.koszalin.pl (K.T.); kornel.pietrzak@s.tu.koszalin.pl (K.P.); lukasz.dudek@tu.koszalin.pl (Ł.D.); 2 Department of Physics, Norwegian University of Science and Technology (NTNU), 7491 Trondheim, Norway; steinar.raaen@ntnu.no; 3 HORIBA FRANCE S.A.S., Boulevard Thomas Gobert - Passage Jobin Yvon, 91120 Palaiseau, France; sofia.gaiaschi@horiba.com (S.G.); patrick.chapon@horiba.com (P.C.); 4 Hochschule Wismar-University of Applied Sciences Technology, Business and Design, Faculty of Engineering, 23966 Wismar, Germany; winfried.malorny@hs-wismar.de; 5 Institute of Geological Engineering, Faculty of Mining and Geology, VŠB—Technical University of Ostrava, 70833 Ostrava, Czech Republic; dalibor.matysek@vsb.cz

**Keywords:** plasma electrolytic oxidation (PEO), micro arc oxidation (MAO), titanium, copper(II) nitrate(V) trihydrate, orthophosphoric acid

## Abstract

The present paper covers the possible ways to fabricate advanced porous coatings that are enriched in copper on a titanium substrate through Direct Current Plasma Electrolytic Oxidation (DC-PEO) with voltage control, in electrolytes made of concentrated orthophosphoric acid with the addition of copper(II) nitrate(V) trihydrate. In these studies, solutions containing from 0 to 650 g salt per 1 dm^3^ of acid and anodic voltages from 450 V up to 650 V were used. The obtained coatings featuring variable porosity could be best defined by the three-dimensional (3D) parameter *Sz*, which lies in the range 9.72 to 45.18 μm. The use of copper(II) nitrate(V) trihydrate in the electrolyte, resulted, for all cases, in the incorporation of the two oxidation forms, i.e., Cu^+^ and Cu^2+^ into the coatings. Detailed X-Ray Photoelectron Spectroscopy (XPS) studies layers allowed for stating that the percentage of copper in the surface layer of the obtained coatings was in the range of 0.24 at% to 2.59 at%. The X-Ray Diffraction (XRD) studies showed the presence of copper (α-Cu_2_P_2_O_7_, and Cu_3_(PO_4_)_2_) and titanium (TiO_2_-anatase, TiO_3_, TiP_2_O_7_, and Ti_0.73_O_0.91_) compounds in coatings. From Energy-Dispersive X-Ray Spectroscopy (EDS) and XPS studies, it was found that the Cu/P ratio increases with the increase of voltage and the amount of salt in the electrolyte. The depth profile analysis by Glow-Discharge Optical Emission Spectroscopy (GDOES) method showed that a three-layer model consisting of a top porous layer, a semi-porous layer, and a transient/barrier layer might describe the fabricated coatings.

## 1. Introduction

The Plasma Electrolytic Oxidation (PEO) process, which is known also under the name of Micro Arc Oxidation (MAO), is used to fabricate porous coatings on light metals substrates, such as titanium and its alloys. The PEO coatings can be used as transition layers between the bones and implants because of their chemical composition. They are also interesting background substrates for catalysts. Previous studies of the authors have proved that it is possible to obtain porous coatings on titanium [1] and its alloys [2,3], which are enriched with selected chemical elements, during the PEO processes under both Direct Current (DC) [1,2,3] or Alternating Current (AC) [4] voltage conditions in electrolytes that are based on orthophosphoric acid. It is important that most of the research teams all over the world involved in the fabrication and characterization of such coatings on pure titanium or on its alloys are using water electrolytes, as shown in Table 1. Most of presented electrolytes, which are based on sodium hexametaphosphate, sodium pyrophosphate, trisodium phosphate, as well as sodium and calcium dihydrogen phosphates with the addition of selected compounds, influenced the improvement of the mechanical and electrochemical properties. It has to be pointed out that additional antibacterial protection can be obtained by fabrication of PEO coatings in solutions mostly containing silver or copper ions, which may be obtained by the use of CH_3_COOAg [5] or Cu(CH_3_COO)_2_ [6], respectively. It was also indicated that thermal treatment essentially affects the surface roughness and its wettability, without affecting the titanium, calcium, and phosphorus oxidation levels that are present in the coating [7]. The surface roughness and its wettability are connected with the biocompatibility of the material and proliferation properties of coatings applied in implants. However, it was pointed out that, for medical applications, the optimization of the oxide surface properties is of extreme importance and a necessity [8]. In addition, increasing the values of roughness parameters might result in a limitation of the adhesion of the cells to the coating, which leads to a reduced ability for proliferation. The effect of the diameter of the titanium sphere structures on the porosity of the PEO coatings and chemical compounds of titanium with oxygen that formed in the obtained coatings was also proved [9].

On the other hand, it is the studies of the mechanical properties of PEO coatings obtained on titanium alloy in aqueous electrolytes with the additions of NaAlO_2_, Na_3_PO_4_, Na_2_SiO_3_, and KOH have proved that using an electrolyte composition of NaAlO_2_ with Na_3_PO_4_ offers considerable improvement of the mechanical properties when compared to the ones of the alloy surface before its modification [10]. The presence of anatase and rutile on the alloy surfaces in an aqueous solution of Na_3_PO_4_ allowed for potential use in photocatalysis [11]. 

In this current paper, we will therefore present some complex multivariate analysis of our coatings involving both the chemical and the phase compositions, together with roughness aspects that are related to the changes of electrolytes’ composition and PEO voltages. We propose also mathematical formulae that will help in predicting this surface roughness as well as the chemical and phase compositions from the parameters of the process – an information that is not attainable so far in the available literature.

## 2. Materials and Methods 

Commercially Pure Titanium Grade 2 samples (1000 mm × 10 mm × 2 mm) were used for all experiments. The preparation procedure and PEO set up on DC Power Supply (Kikusui PWR 1600H, Kikusui Electronics Corporation, Yokohama, Kanagawa, Japan) as well as the coatings’ characterization methods, such as: SEM (FEI Quanta 250, Field Electron and Iron Company, Hillsboro, OR, USA), EDS (ultra-dry EDS detector of Thermo Scientific Co, Madison, WI, USA), XRD (D8 Advance System of Bruker’s Co, BRUKER Corporation, Billerica, MA, USA), XPS (SCIENCE SES 2002, ScientaOmicron, Uppsala, Sweden), GDOES (Horiba Profiler 2, HORIBA Scientific, Palaiseau, France), and Confocal Laser Microscope (LEXT OLS 4000, Olympus Co, Tokyo, Japan), are described in detail in the Supplement (Section 1) and reference [25]. 

## 3. Results

### 3.1. Scanning Electron Microscopy and Confocal Laser Scanning Microscopy Studies

Scanning electron microscopy (SEM) and confocal laser scanning microscopy (CLSM) were used to view and describe the surfaces of porous samples after PEO treatment and to analyze their surface geometry structure. The topography of the surfaces is given by the three-dimensional (3D) parameters describing roughness (*Sp*, *Sv*, *Sz*, *Sa*), as given by CLSM. The measurements of the surface geometrical structure were independently carried out on three planes in each point of the research plan. 

**Figure 1:**
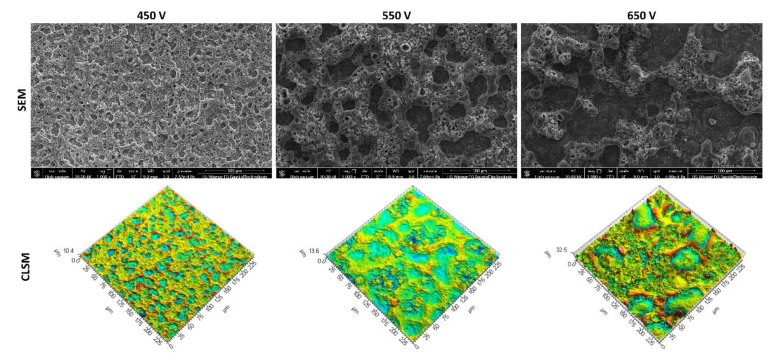
Exemplary scanning electron microscopy (SEM) images and three-dimensional (3D) maps obtained by using confocal laser scanning microscopy (CLSM) technique on the Plasma Electrolytic Oxidation (PEO) coatings fabricated with an electrolyte of pure concentrated orthophosphoric acid at voltages: 450 V, 550 V, and 650 V.

**Figure 2:**
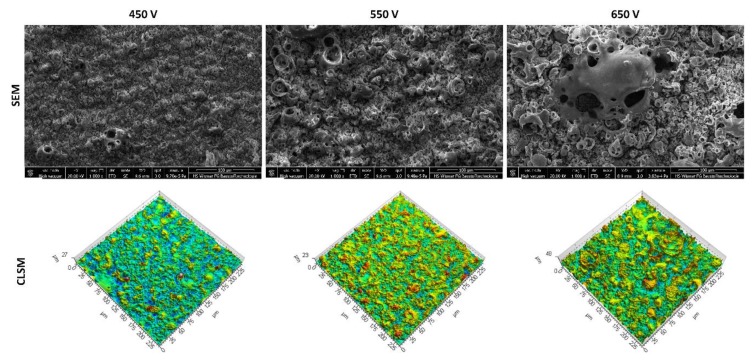
Exemplary SEM images and 3D maps obtained by using CLSM technique on the PEO coatings fabricated with an electrolyte of concentrated orthophosphoric acid with copper(II) nitrate trihydrate (650 g/dm^3^) at voltages: 450 V, 550 V, and 650 V.

Various electrolytes of orthophosphoric acid and orthophosphoric acid with the addition of the copper (II) nitrate (V) trihydrate in the following amounts: 10, 50, 200, 350, 500, and 650 g/dm^3^ were used and the PEO processes were carried out under three voltages: 450, 550, and 650 V to determine the effect of voltage in the PEO process. All of the surfaces were viewed by SEM and CLSM and all of them are presented in Figures S1–S7 and the representative examples of those obtained in the electrolyte consisting of concentrated (85 wt%) orthophosphoric acid without and with copper(II) nitrate trihydrate, which are presented below in Figure 1 and Figure 2, respectively.

The SEM images of the coatings display the porous surfaces, on which the “pores-in-pores” may be observed. The coating obtained at 650 V shows the occurrence of cracks in its structure, as can be seen in Figure S1i,l, which is an undesired negative effect. 

CLSM was utilized to study the influence of the process voltage on the change of the surface geometrical structure, with the results being presented by means of 3D maps. CLSM allowed for determining the parameters for the evaluation of the surface roughness; on the basis of these results, the parameters characterizing the surface topography, together with their basic statistical measures are determined, and shown in Table S1. The non-parametric ranks’ sum test was carried out with the assumed significance level *α* = 0.05 to determine the significance of the effect of the voltage on the parameters for the evaluation of 3D surface roughness. As a result, it was stated that increasing the voltage from 450 V up to 550 V has no essential effect on the parameters *Sp*, *Sv*, and *Sz*, at the assumed significance level *α* = 0.05. *Sa* is the only parameter that allows for differentiating the obtained surfaces. The average value of *Sa* for the surface obtained at the voltage 450 V is equal to 1.13 μm and falls down to 1.03 μm for the coating that was obtained at the voltage of 550 V. 

Between 550 V and 650 V, however, all of the mentioned parameters show significant variation. The increase of voltage from 550 V up to 650 V resulted in an increase of the average value of the roughness parameters *Sp* (from 6.29 μm to 15.20 μm), *Sv* (from 6.78 μm to 16.37 μm), *Sz* (from 13.08 μm to 31.54 μm), and *Sa* (from 1.03 μm to 2.87 μm). 

In Figure S2, the SEM images and 3D maps of the surface geometrical structure, characterizing the surface of coatings that were obtained in an electrolyte consisting of 10 g of the copper(II) nitrate(V) trihydrate and 1 dm^3^ of orthophosphoric acid, are shown. Alike the coatings that were obtained in concentrated orthophosphoric acid, without any salt used, the increase of the voltage in the PEO process from 450 V to 650 V resulted in the change of pores stereometry, which can be observed in Figure S2. Each of the obtained surfaces is characteristic with the “pores-in-pores”, which are shown in Figure S2g–l. The 3D surfaces, which were obtained on the basis of CLSM, as presented in Figure S2m–o, confirm the results of SEM imaging. From the CLSM results, the parameters for the evaluation of the 3D surface roughness were determined, with the magnitudes and statistical measures that are given in Table S2. The increase of voltage in the PEO process from 450 V to 550 V resulted in the increase of average values of all parameters *Sp* (from 6.24 μm to 12.57 μm), *Sv* (from 5.51 μm to 12.37 μm), *Sz* (from 11.75 μm to 24.94 μm), and *Sa* (from 1.00 to 2.35 μm). This trend continues when the voltage increases from 550 V to 650 V. The surfaces of porous coatings that were obtained in the electrolyte containing 50 g Cu(NO_3_)_2_∙3H_2_O in 1 dm^3^ H_3_PO_4_ at the voltages of 450 V, 550 V, and 650 V are illustrated by SEM images (Figure S3a–l), and the CLSM results (Figure S3m–o). In the SEM images that are presented in Figure S3g–l, one might observe the occurrence of “pores-in-pores” characteristic phenomena of the obtained surface. The 3D surface roughness parameters have been assigned and are presented in Table S3 and studied for the significance, as previously explained. An increase of the voltage of the PEO process from 450 V up to 550 V resulted in the increase of average values of all the roughness parameters *Sp* (from 4.41 μm to 13.44 μm), *Sv* (from 5.31 μm to 16.22 μm), *Sz* (from 9.72 μm to 29.67 μm), and *Sa* (from 0.99 μm to 2.87 μm). 

However, when the voltages ranged from 550 V to 650 V, we observe a significant change of *Sv* and *Sa*, with their average value getting down from 16.22 μm down to 12.00 μm for *Sv*, and from 2.87 μm to 2.00 μm for *Sa*. 

The next step in the evaluation of the effect of the content of copper(II) nitrate(V) trihydrate in orthophosphoric acid and the voltage on the surface geometry of the porous coatings was pursued with electrolyte containing 200 g Cu(NO_3_)∙3H_2_O in 1 dm^3^ H_3_PO_4_ at voltages of 450 V, 550 V, and 650 V. The results obtained in the form of SEM images and 3D models have been correlated in Figure S4. Similarly to the cases described before, changes in the development of the porous surface are linked to the increase of voltage for the PEO process, with the results that are presented in Figure S4a–e. At 650 V, inside the pores visible on the surface of the coating, there are “pores-in-pores” clearly visible. This phenomenon is more pronounced than in the case of coatings obtained in electrolytes with lower contents of salt, as presented in Figure S4i. The surfaces of the obtained coatings in this case, at each of the voltages applied, feature more developed stereometric structure, as shown in Figure S4j–l. The observed changes in the pictures that were obtained by SEM technique have been confirmed by the results obtained while the using CLSM technique, as presented in Figure S4m–o. Table S4 summarizes the obtained results.

The increase in voltages from 450 V to 550 V resulted in an essential growth of the average values of all parameters, at the assumed significance level of *α* = 0.05. However, from 550 V to 650 V, only the *Sz* parameter shows a significant increase (again at the assumed significance level *α* = 0.05). Figure S5 displays the surfaces of the porous coatings obtained in the electrolyte consisting of 350 g of copper(II) nitrate(V) trihydrate dissolved in 1 dm^3^ of orthophosphoric acid at voltages of 450 V, 550 V, and 650 V. Based on the analysis of SEM images, one may state that pores occurring in the coatings that were obtained under the voltages of 450 V, and 550 V (Figure S5a–b) are characteristic with a different stereometry, whereas the surfaces obtained at voltages of 550 V and 650 V may be qualified as very similar, as seen in Figure S5n–o. The CLSM measurements for quantitatively describing the parameters to evaluate the surface roughness are shown in Figure S5m–n, and collected in Table S5. The significance test displays a great effect of voltage change from 450 V to 550 V for all parameters *Sp*, *Sv*, *Sz*, and *Sa* with the average values increasing by 9.32 μm for *Sp*, 12.20 μm for *Sv*, 21.53 μm for *Sz*, and 1.50 μm for *Sa*. However, the same tests for voltages between 550 V and 650 V do not reveal significant differences. 

The following results that are presented in Figure S6 concern the PEO coatings obtained in an electrolyte containing 500 g Cu(NO_3_)_2_∙3H_2_O in 1 dm^3^ H_3_PO_4_ at voltages of 450 V, 550 V, and 650 V. The increase in the amount of salt leads to coatings that have a different outer structure as compared to the ones previously observed and the SEM images do not univocally allow for differentiating the surfaces, as seen in Figure S6g–l. For this reason, to describe the surface topography, the roughness parameters to evaluate 3D surfaces, determined on the basis of the performed CLSM studies, were used. Table S6 collects the determined parameters, together with their basic statistical measures and significance. The change of voltage from 450 V to 550 V resulted in a substantial increase of values of all parameters *Sp* (from 8.66 μm to 14.20 μm), *Sv* (from 8.70 μm to 15.96 μm), *Sz* (from 17.36 μm to 30.16 μm), and *Sa* (from 1,33 μm to 2.32 μm). The significance studies of the effect of voltage change of the PEO process from 550 V to 650 V on the parameters serving to evaluate the 3D surface roughness have been also performed, and some parameters feature a substantial growth *Sp* (from 14.20 μm to 22.61 μm), *Sv* (from 15.96 μm to 22.57 μm), and *Sa* (from 2.32 μm to 3.42 μm), whereas *Sz* does not change significantly. 

The highest content of copper(II) nitrate(V) trihydrate dissolved in 1 dm^3^ of orthophosphoric acid, for which it was possible to obtain the porous PEO coatings at all voltages 450 V, 550 V, and 650 V, is equal to 650 g. The study results of the surfaces that were obtained with such amount of salt, have been placed in Figure S7 and partly in Figure 2 of this paper. Table S7 collects the 3D surface roughness parameters. No significant differences occur between 450 V and 550 V, but between 550 V and 650 V there are essential differences for *Sp* (increase of the average value from 12.74 μm to 20.58 μm) and *Sa* (increase of the average value from 2.09 μm to 3.59 μm), whereas *Sv* and *Sz* do not statistically differ. 

### 3.2. EDS Studies

For a statistical quantitative description of the elementary composition of the PEO coatings that were obtained in the electrolyte made of concentrated orthophosphoric acid and copper(II) nitrate(V) trihydrate, the EDS technique was used. In order to determine the atomic ratio Cu/P, a study plan taking into account the detection/quantification limit values of copper by the EDS method was employed and quantitative EDS studies were carried out for the PEO coatings that were obtained in electrolytes containing significant amount of Cu(NO_3_)_2_∙3H_2_O, namely 200 g, 350 g, 500 g, and 650 g in 1 dm^3^ H_3_PO_4_ at all voltages 450 V, 550 V, and 650 V. 

**Figure 3:**
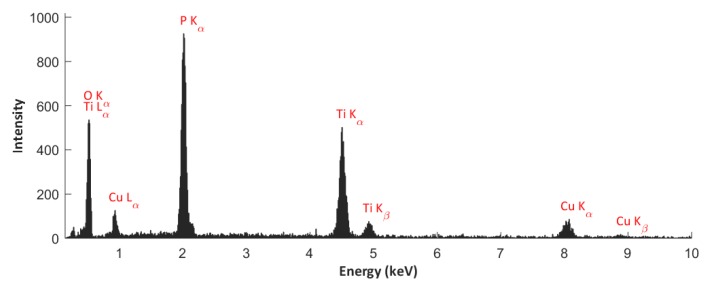
Example of EDS spectrum obtained on the surface of a titanium sample after the PEO treatment in the electrolyte containing 500 g Cu(NO_3_)_2_∙3H_2_O in 1 dm^3^ H_3_PO_4_ at the voltage of 550 V.

All of the EDS studies were carried out on ten surfaces of the titanium samples after the PEO process, with ten repetitions on each of the surfaces with the magnification used equaling 500×, resulting, in total, 100 repetitions for one point of the study plan. Figure 3 provides an exemplary EDS spectrum.

**Figure 4:**
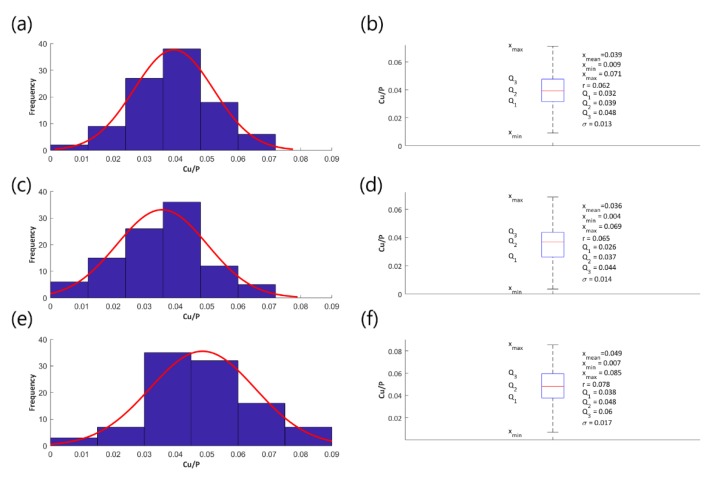
Histograms of the atomic ratio Cu/P (**a**,**c**,**e**), box plots and basic statistical measures (**b**,**d**,**f**) for the coatings that were obtained in the electrolyte consisting of 200 g/dm^3^ of Cu(NO_3_)_2_∙3H_2_O at the voltages 450 V (**a**,**b**), 550 V (**c**,**d**), and 650 V (**e**,**f**).

**Figure 5:**
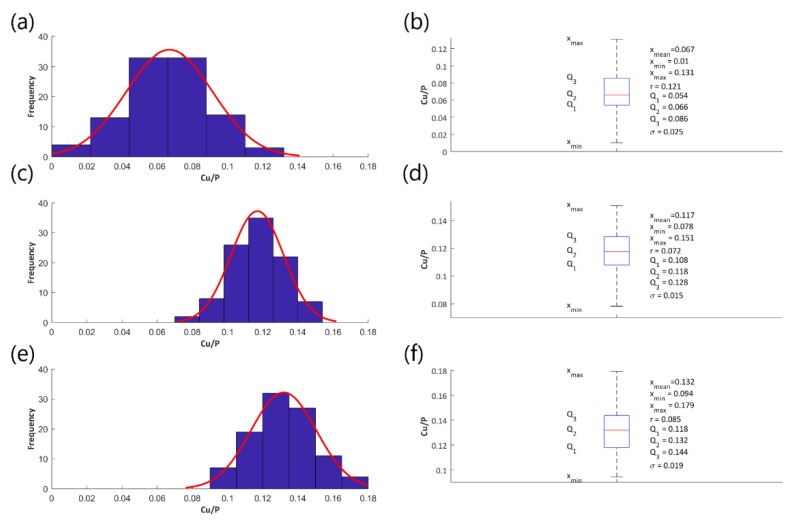
Histograms of the atomic ratio Cu/P (**a**,**c**,**e**), box plots and basic statistical measures (**b**,**d**,**f**) for the coatings obtained in the electrolyte consisting of 350 g/dm^3^ of Cu(NO3)_2_∙3H_2_O at the voltages 450 V (**a**,**b**), 550 V (**c**,**d**), and 650 V (**e**,**f**).

**Figure 6:**
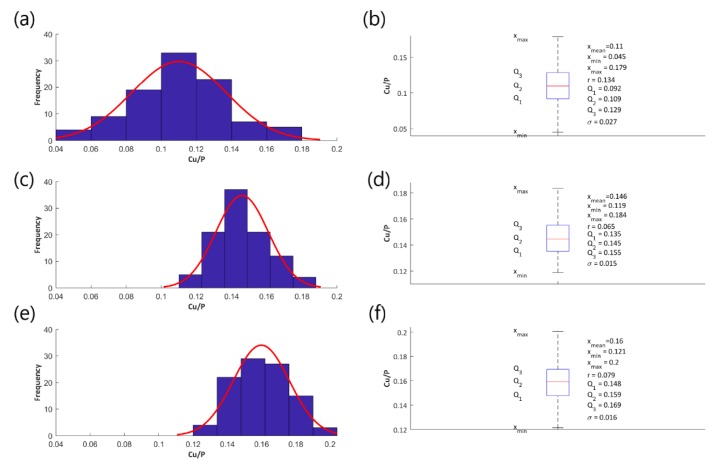
Histograms of the atomic ratio Cu/P (**a**,**c**,**e**), box plots and the basic statistical measures (**b**,**d**,**f**) for the coatings obtained in the electrolyte consisting of Cu(NO_3_)_2_∙3H_2_O amounting for 500 g/dm^3^ at the voltages 450 V (**a**,**b**), 550 V (**c**,**d**), and 650 V (**e**,**f**).

**Figure 7:**
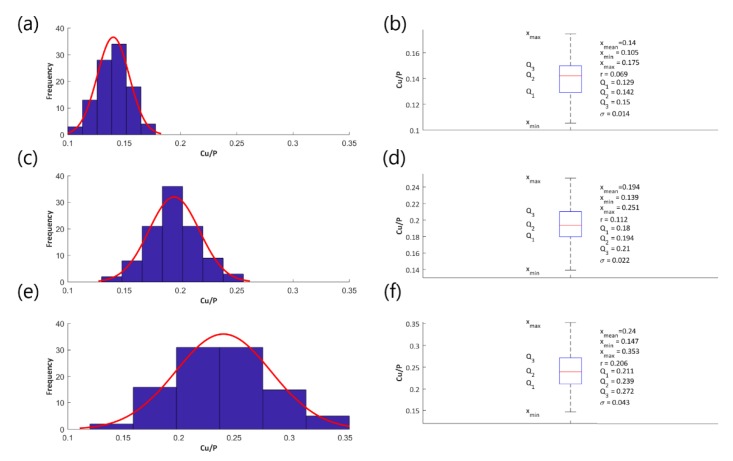
Histograms of the atomic ratio Cu/P (**a**,**c**,**e**), box plots and basic statistical measures (**b**,**d**,**f**) for the coatings obtained in the electrolyte consisting of 650 g/dm^3^ of Cu(NO_3_)_2_∙3H_2_O at the voltages 450 V (**a**,**b**), 550 V (**c**,**d**), and 650 V (**e**,**f**).

Histograms and fitted normal distributions, as well as basic statistical measures of the determined atomic ratios Cu/P, have been established to illustrate the effect of the voltage change on the parameter Cu/P and they are shown in the following Figure 4 (coatings obtained in the electrolyte containing 200 g Cu(NO_3_)_2_∙3H_2_O in 1 dm^3^ H_3_PO_4_), Figure 5 (coatings obtained in the electrolyte containing 350 g Cu(NO_3_)_2_∙3H_2_O in 1 dm^3^ H_3_PO_4_), and Figure 6 (coatings obtained in the electrolyte containing 500 g Cu(NO_3_)_2_∙3H_2_O in 1 dm^3^ H_3_PO_4_), Figure 7 (coatings obtained in the electrolyte containing 650 g Cu(NO_3_)_2_∙3H_2_O in 1 dm^3^ H_3_PO_4_). 

Figure 4a,c,e, Figure 5a,c,e, Figure 6a,c,e, and Figure 7a,c,e represent the histograms with the normal distributions (red lines) for each of the voltages 450 V, 550 V, and 650 V, and Figure 4b,d,f, Figure 5b,d,f, Figure 6b,d,f, and Figure 7b,d,f show the box plots with statistical measures for the corresponding voltages. 

### 3.3. XPS Studies

XPS studies were carried out, with the qualitative and quantitative analyses of all the XPS data presented in Figures S8–S16 to describe the chemical composition of the top 10-nm surface layer of the PEO coatings; an exemplary one with maximum amount of copper inside has been presented below, as in Figure 8. 

The compounds of titanium and copper are present, as proved by the binding energies in the ranges of 459.4–460.8 eV (Ti^4+^) and 932.2–936.8 eV (Cu^2+^, and/or Cu^+^). On the other hand, the analyses of spectra of phosphorus P 2p (133.6–135.4 eV) and oxygen O 1s (531.1–532.7 eV) indicate the presence of phosphates, and/or diphosphates, and/or phosphates groups. Moreover, the position of the highest peak value in the spectrum of O 1s does not exclude the presence of hydroxyl groups, and metal oxides in the surface layer. The hydroxyl groups may be connected in the coating in the form of hydroxyphosphates and/or hydroxyoxides. 

The XPS calculations reveal the occurrence of non-stoichiometric phosphate groups. The analyses of C 1s (284.8 eV) and N 1s (399.6–403.4 eV) spectra indicate the presence of both carbon and nitrogen that result from atmospheric contamination and possibly introduced during the preparation of the samples. The N 1s spectrum might also indicate the presence of ammonia compounds. The increase of the amount of the copper(II) nitrate(V) in the electrolyte from 50 to 500 g/dm^3^ at a constant voltage 550 V resulted in the increase of the amount of copper built in the structure of coating from 0.24 to 1.38 at% (over five-fold growth), whereas, at a constant amount of salt in the electrolyte (500 g/dm^3^), a change in the process voltage from 450 V to 650 V results in an increase of the amount of copper from 1.18 to 1.81 at% (over 1.5-fold growth). If the amount of salt in the solution is up to 650 g/dm^3^, the same increase of voltage from 450 V to 650 V resulted in an amount of copper in the coating to reach 2.33–2.59 at%. In addition, the ratio Cu/P was in the interval of 0.009 to 0.082.

### 3.4. XRD Studies

**Figure 9:**
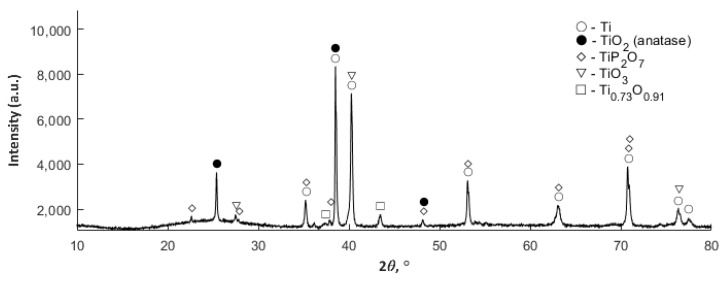
XRD diffractogram obtained for the PEO coating fabricated in the electrolyte consisting of orthophosphoric acid and copper(II) nitrate(V) trihydrate at concentration 50 g/dm^3^ and voltage of 550 V.

XRD studies were also carried out in agreement with the assumed study plan, and the results have been displayed in Figures S17–S24. The coatings obtained in the electrolytes with 10 g/dm^3^ Cu(NO_3_)_2_∙3H_2_O (Figure S17) and 50 g/dm^3^ (Figure 9 in the paper) at a voltage of 550 V, containing in their structure the titanium oxides (TiO_2_ (anatase), TiO_3_, Ti_0,73_O_0,91_) and diphosphate(V) of titanium(IV) (TiP_2_O_7_), in the crystal form.

Increasing the content of salt Cu(NO_3_)_2_∙3H_2_O from 10 to 50 g/dm^3^ resulted in an increase of the observed halo 2*ϴ* in the range of 20° to 30°, which is most probably connected with the formation of an amorphous or nano-crystalline phase. The coatings that were obtained in electrolytes with 200 g Cu(NO_3_)_2_∙3H_2_O (Figure S19) and 350 g Cu(NO_3_)_2_∙3H_2_O (Figure S20) in 1 dm^3^ H_3_PO_4_ at a voltage of 550 V show the presence of anatase (TiO_2_) and titanium(IV) diphosphate(V) (TiP_2_O_7_); here also the increase of the content of copper(II) nitrate(V) trihydrate in the electrolyte leads to an increase of the intensity of the observed halo 2ϴ in the range from 20° to 30°. 

The XRD diffractogram that was obtained for the PEO coating fabricated in the electrolyte containing 500 g/dm^3^ Cu(NO_3_)_2_∙3H_2_O at the voltage of 550 V (Figure S21) indicated the presence of titanium only with a halo for 2ϴ in the range from 20° to 30°. In these conditions, the PEO coating is most probably nano-structured or it has an amorphous structure. 

**Figure 10:**
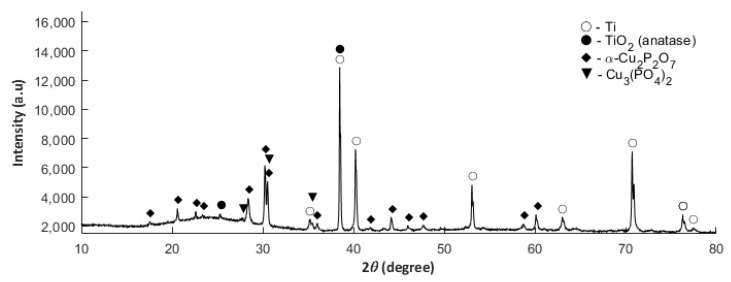
XRD diffractogram obtained for the PEO coating fabricated in the electrolyte consisting of orthophosphoric acid and the copper(II) nitrate(V) trihydrate of concentration 650 g/dm^3^ and voltage of 550 V.

We have decided to vary the PEO process potential/voltage from 450 V to 650 V, keeping a constant chemical composition of the electrolyte (500 g Cu(NO_3_)_2_∙3H_2_O in 1 dm^3^ H_3_PO_4_), in order to study the effect of the voltage on the formation of a crystalline phase identifiable by XRD. The XRD results proved that the decrease of the potential/voltage of the PEO process from 550 to 450 V leads to the detection in the crystalline phase of anatase (TiO_2_) and titanium(IV) and titanium(IV) diphosphate(V) (TiP_2_O_7_), (TiP_2_O_7_), which are displayed in Figure S22, whereas the increase of voltage up to 650 V resulted in the recording of a crystalline phase, consisting of the anatase (TiO_2_) and titanium(IV) diphosphate(V) (TiP_2_O_7_), (Figure S23). The increase of the contents of Cu(NO_3_)_2_∙3H_2_O in the electrolyte up to 650 g/dm^3^ at a fixed voltage 550 V resulted in identifying the anatase (TiO_2_) and the compounds of copper: α– copper(II) diphosphate(V) (Cu_2_P_2_O_7_) and copper(II) phosphate(V) (Cu_3_(PO_4_)_2_), as displayed below, as in Figure 10.

### 3.5. GDOES Studies

**Figure 11:**
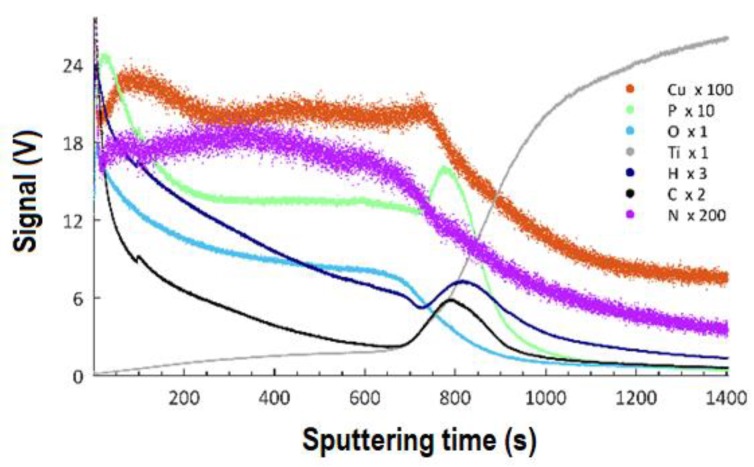
GDOES depth profile of coating obtained in the electrolyte containing 350 g/dm^3^ of Cu(NO)_3_∙3H_2_O with PEO voltage of 450 V. The elements Cu, P, O, Ti, H, C, and N may be present in the coatings or result from adsorbed contamination from the atmosphere or cleaning procedure.

**Figure 12:**
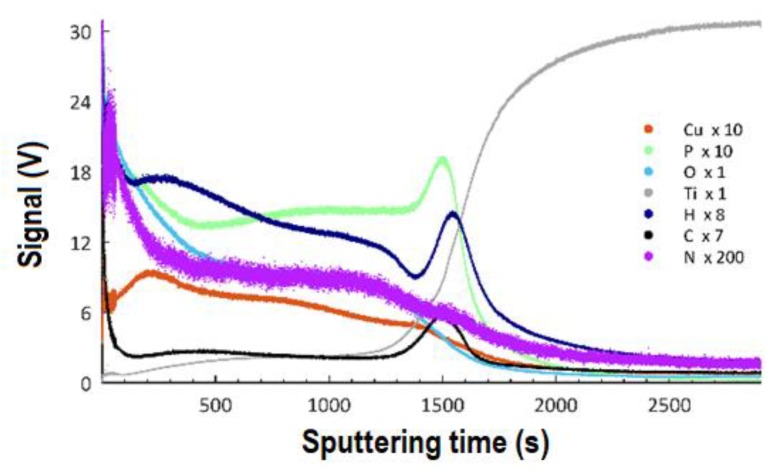
GDOES depth profile of coating obtained in the electrolyte containing 350 g/dm^3^ Cu(NO)_3_∙3H_2_O with PEO voltage of 550 V. The elements Cu, P, O, Ti, H, C, and N may be present in the coatings or result from adsorbed contamination from the atmosphere or cleaning procedure.

**Figure 13:**
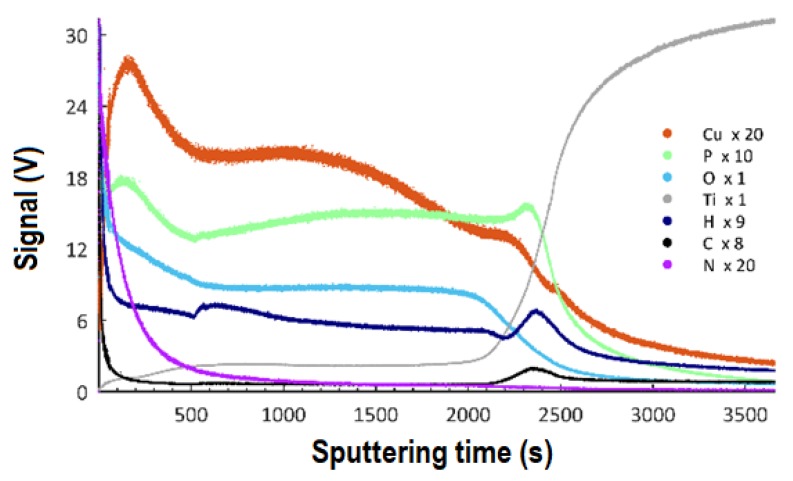
GDOES depth profile of coating obtained in the electrolyte containing 350 g/dm^3^ Cu(NO)_3_∙3H_2_O with PEO voltage of 650 V. The elements Cu, P, O, Ti, H, C, and N may be present in the coatings or result from adsorbed contamination from the atmosphere or cleaning procedure.

The Glow-Discharge Optical Emission Spectroscopy (GDOES) technique was used to determine the effect of the applied voltage in the PEO process on the thicknesses of the obtained coatings and the distribution of particular elements as a function of depth. The GDOES measurements were carried out on the samples after PEO treatment with an electrolyte containing 350 g of the copper(II) nitrate(V) trihydrate in 1 dm^3^ of orthophosphoric (85 wt%) acid and at voltages of 450 V, 550 V, and 650 V, and the results are presented in Figure 11 (450 V), Figure 12 (550 V), and Figure 13 (650 V). 

The results allow for stating that the coatings obtained at all voltages may be described as three consecutive layers: a porous layer, a semi-porous layer, and a transient layer. The outer porous layer is enriched with copper, phosphorus, oxygen, hydrogen, carbon, and nitrogen, but depleted in titanium. In each of the analyzed coatings, the characteristic feature of this outer layer is the occurrence of a global maximum for the signals of: copper, phosphorus, oxygen, hydrogen, carbon, and nitrogen. The determined thickness of the porous layers for the samples that were obtained at the voltages of 450 V, 550 V, and 650 V was, as follows: 1.9 μm (220 s of GDOES etching time), 3.5 μm (400 s of GDOES etching time), and 4.3 μm (500 s of GDOES etching time). The porous layer is the thinner one of the three considered layers of the PEO coating. The next layer, placed under the porous one, is semi-porous and featured by signals of phosphorus, oxygen, nitrogen, hydrogen, and carbon close to a plateau. The copper signal for this layer (in the case of a coating fabricated at the voltage of 450 V) is almost constant in depth, whereas a decreasing trend for Cu is observed for higher voltages (550 V, 650 V). 

The determined depths of the semi-porous layer are approximately 4.0 μm for 450 V, 7.8 μm for 550 V, and 13.9 μm for 650 V. The most inner layer of the PEO coatings is a transient layer, connecting the semi-porous layer with the titanium substrate. This transient layer has local maxima for phosphorus, hydrogen, and carbon, after which a fall of the intensity of the corresponding signals is observed. For each of the samples fabricated in the electrolyte containing copper(II) nitrate(V) trihydrate at 350 g/dm^3^, the fall of signals of copper, oxygen, and nitrogen is detected, coinciding with the rise of the titanium signal. The determined thicknesses of the transient layers for the samples that were obtained at the voltages of 450 V, 550 V, and 650 V, were about 3.7 μm, 8.7 μm, and 9.6 μm, respectively. Summing up, the thicknesses of the different sub-layers identified the total thickness of the obtained porous PEO coatings, containing copper, in the electrolyte consisting of 350 g Cu(NO_3_)∙3H_2_O were, as follows: 9.6 μm (for 450 V), 20.0 μm (for 550 V), and 27.8 μm (for 650 V).

## 4. Discussion

**Figure 14:**
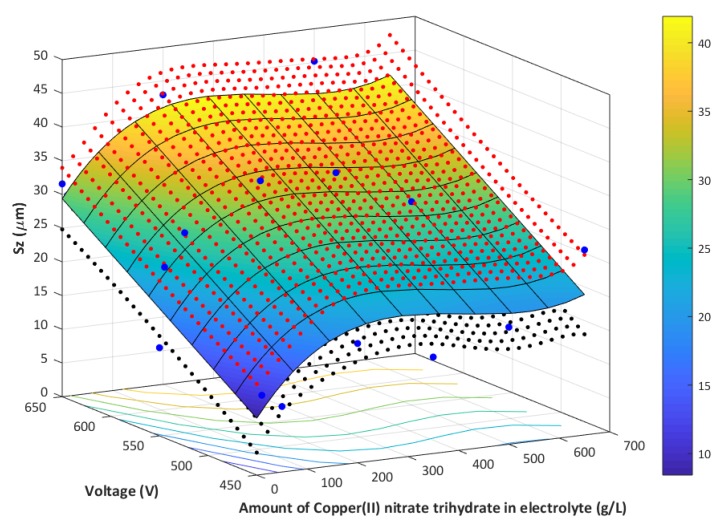
Graphical representation of the mathematical model (blue–yellow gradient), together with the confidence intervals of the model (red points – upper range, blue points – lower range). Sz as a function of the contents of Cu(NO_3_)_2_∙3H_2_O in 1 dm^3^ H_3_PO_4_ and at varying voltages; the confidence interval is the statistical space (α = 0.05).

Based on the obtained results on surface roughness, it was clear that the parameter *Sz* might be used as the best representative to describe the porous coatings that were obtained on titanium after plasma electrolytic oxidation. An increase of the PEO voltage results in an increase of the *Sz* value, as observed on the 3D maps that were analyzed while using the CLSM technique. An additive model was assumed in order to best describe the effect of the content of copper(II) nitrate(V) trihydrate in the electrolyte on *Sz* at varying voltages of 450 V, 550 V, and 650 V, which is summarized in Equation (1) and graphically presented in Figure 14.

Y = 2.3 × 10^−7^ × x_1_^3^ − 2.83 × 10^−4^ × x_1_^2^ + 0,106 × x_1_ + 0.10 × x_2_ − 38.7 (1)

where *x_1_* is the content of the copper(II) nitrate(V) trihydrate in 1 dm^3^ of orthophosphoric acid, and it is expressed in g/dm^3^, and *x_2_* denotes the voltage of the PEO process expressed in volts—moreover x_1_ ∈ 〈0─650〉 and x_2_ ∈ 〈450─650〉.

**Figure 15:**
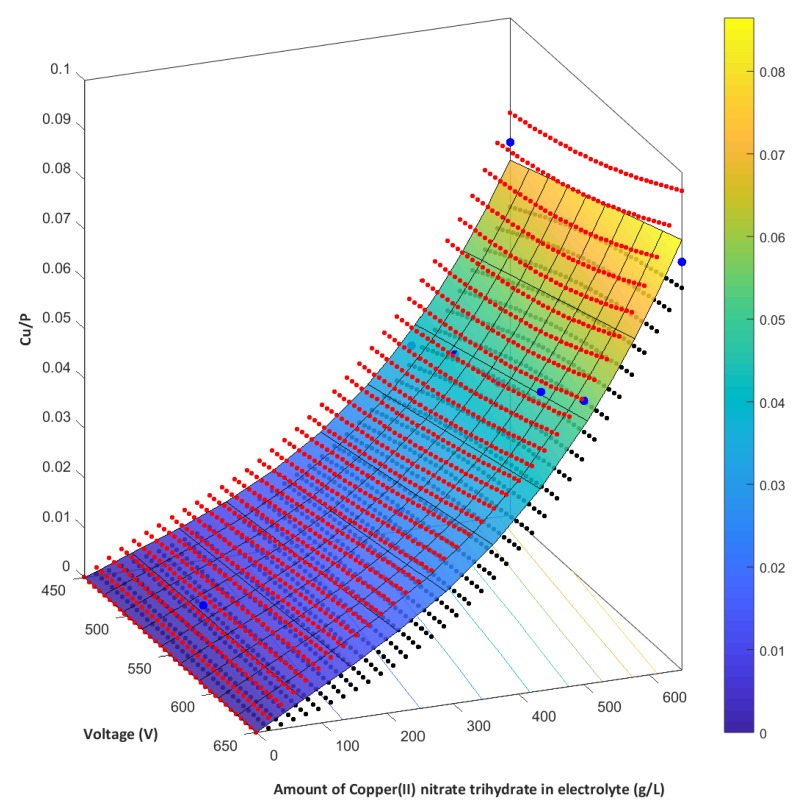
Graphical representation of the mathematical model (blue–yellow gradient), together with the confidence intervals of the model (red points—upper range, blue points—lower range) designated on the basis of the XPS analyses and describing the atomic ratio Cu/P (blue points) as a function of the content of Cu(NO_3_)_2_∙3H_2_O in 1 dm^3^ H_3_PO_4_ and the PEO voltage; the confidence interval is the statistical space (α = 0.05).

We also propose a model for the variations of the chemical composition of the top 10 nm surface (measured from XPS data) as a function of the atomic ratio Cu/P and the amount of the copper(II) nitrate(V) trihydrate in 1 dm^3^ of orthophosphoric acid for different voltages. This model is a multinomial one, as described by the Equation (2):

Y = 2.15 × 10^−10^ × x_1_^3^ − 5.07 × 10^−8^ × x_1_^2^ + 1.16 × 10^−7^ × x_1_ + 0.10 × x_1_ × x_2_ (2)

where *x_1_* is the content of the copper(II) nitrate(V) trihydrate in 1 dm^3^ of orthophosphoric acid, and it is expressed in g/dm^3^, and *x_2_* denotes the voltage of the PEO process expressed in volts—moreover x_1_ ∈ 〈0─650〉 and x_2_ ∈ 〈450─650〉. Figure 15 illustrates this model. The multinomial correlation (*R^2^*) equals 0.901, indicating a relatively good fit. 

**Figure 16:**
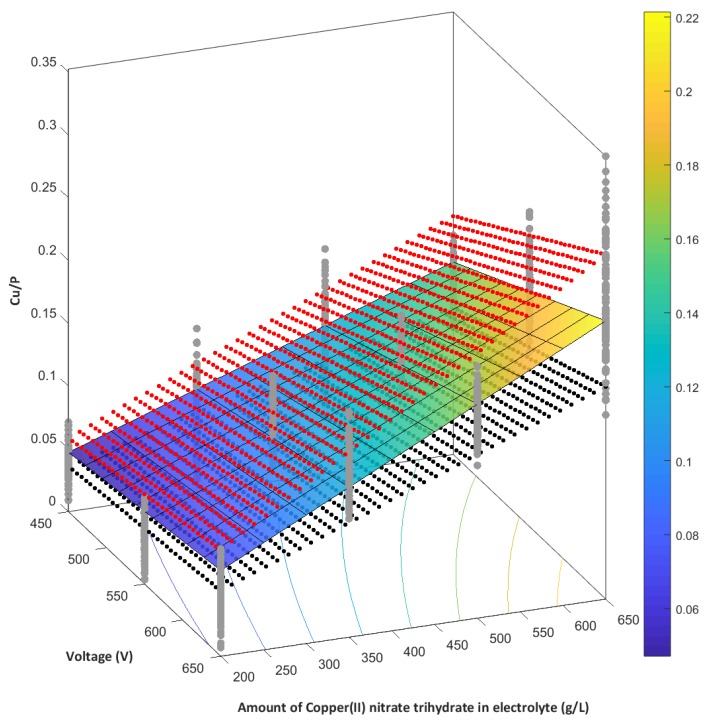
Graphical representation of the mathematical model (blue–yellow gradient), with confidence intervals (red points—upper range, black points—lower range) established from EDS in the second stage of the studies describing the atomic ratio Cu/P (blue points) as a function of the content of Cu(NO_3_)_2_∙3H_2_O in 1 dm^3^ H_3_PO_4_ and the PEO voltage; the confidence interval is the statistical space (α = 0.05).

The EDS results, which characterize the chemical composition of the fabricated PEO coatings, served to establish a mathematical model, characterizing the dependence between the atomic ratio Cu/P and the amount of copper(II) nitrate(V) trihydrate in 1 dm^3^ of orthophosphoric acid and the voltage of the PEO process. Equation (3) describes the model (*R^2^* = 0.843) and it is displayed in Figure 16.

Y = 5.24 × 10^−7^ × x_1_ × x_2_ (3)

where *x_1_* is the contents of the copper(II) nitrate(V) trihydrate in 1 dm^3^ of orthophosphoric acid and it is expressed in g/dm^3^, *x_2_* is the voltage of the PEO process, expressed in volts, with x_1_ ∈ 〈200─650〉 and x_2_ ∈ 〈450─650〉. 

**Figure 17:**
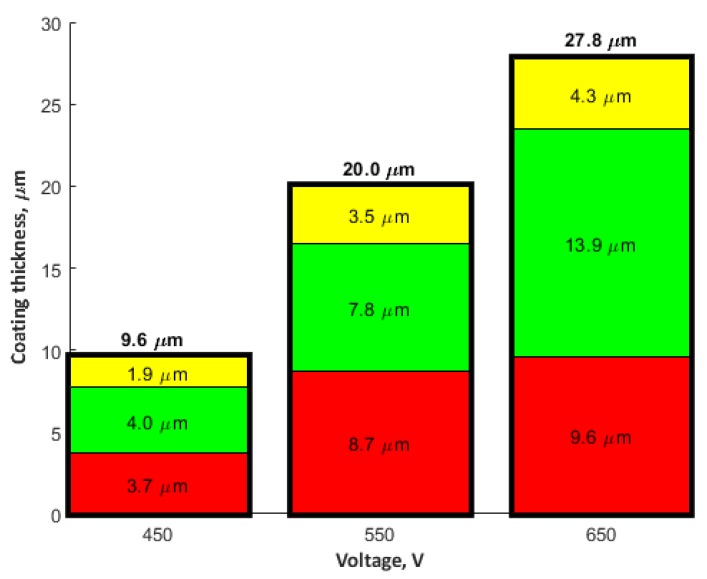
Thickness of the porous layers in the PEO coatings designated on the basis of the GDOES analysis; yellow color—porous layer, green color—semi-porous layer, and red color—transient layer.

From the GDOES studies (Figure 17), a three-layer model has been proposed, which describes the influence of the PEO voltage on the thicknesses of the different layers of the obtained porous coatings.

A voltage increase in the PEO process from 450 V up to 650 V in the same electrolyte (350 g Cu(NO_3_)_2_∙3H_2_O) results in the growth of the total thickness of the obtained coatings from 9.6 µm up to 27.8 µm. Moreover, one should note that the thickness of each of the designated layers (porous, semi-porous, and a transient one) also increases together with the growth of voltage of the PEO process, with the highest increase being observed in the case of the semi-porous layer. 

## 5. Conclusions

Based on the results that are presented in the present paper it can be concluded that it is possible to fabricate porous coatings enriched in copper, with voltages from 450 V to 650 V in the PEO process under DC conditions, in electrolytes based on concentrated orthophosphoric acid with the addition of copper(II) nitrate(V) trihydrate. The following main conclusions may be formulated:

(a) In the obtained PEO coatings, one may distinguish three layers: porous (i), semi-porous (ii), and a transient one (iii); The porous layers are approximately two times thinner than the semi-porous and the transient ones due the fact that the damaging discharges mostly affect the porous layer.
(b) The higher the voltage, the higher the amount of copper in PEO coating.
(c) The higher the amount of Cu(NO_3_)_2_∙3H_2_O used, the higher the amount of copper in PEO coating.
(d) The top (10 nm) of PEO coatings contain Ti^4+^, Cu^+^, and Cu^2+^, as well as PO_4_^3–^, and/or HPO_4_^2–^, and/or H_2_PO_4_^–^, and/or P_2_O_7_^2–^.
(e) The maximum atomic concentration of copper (based on XPS results) in the top surface of PEO coatings equaled 2.59% for the process that was performed in electrolyte consisting of orthophosphoric acid and the copper(II) nitrate(V) trihydrate of concentration 650 g/dm^3^ and voltage of 650 V.
(f) The Cu/P ratios (based on XPS results) on the obtained samples, fabricated in electrolyte with 0-650 g/dm^3^ Cu(NO_3_)_2_ and voltages between 450 V and 650 V, are in the range of 0.009 up to 0.082.
(g) The 3D roughness parameters (Sz/Sa) of the obtained samples, fabricated in electrolyte with 0–650 g/dm^3^ Cu(NO_3_)_2_ and voltages between 450 V and 650 V, are in the range of 9.72 μm/0.99 μm, up to 45.18 μm/3.42 μm.
(h) The thicknesses of PEO coatings, obtained in electrolyte with 350 g/dm^3^ Cu(NO_3_)_2_ and at voltages between 450V and 650 V, are in the range from 9.6 μm up to 27.8 μm.

## Figures and Tables

**Figure 8:**
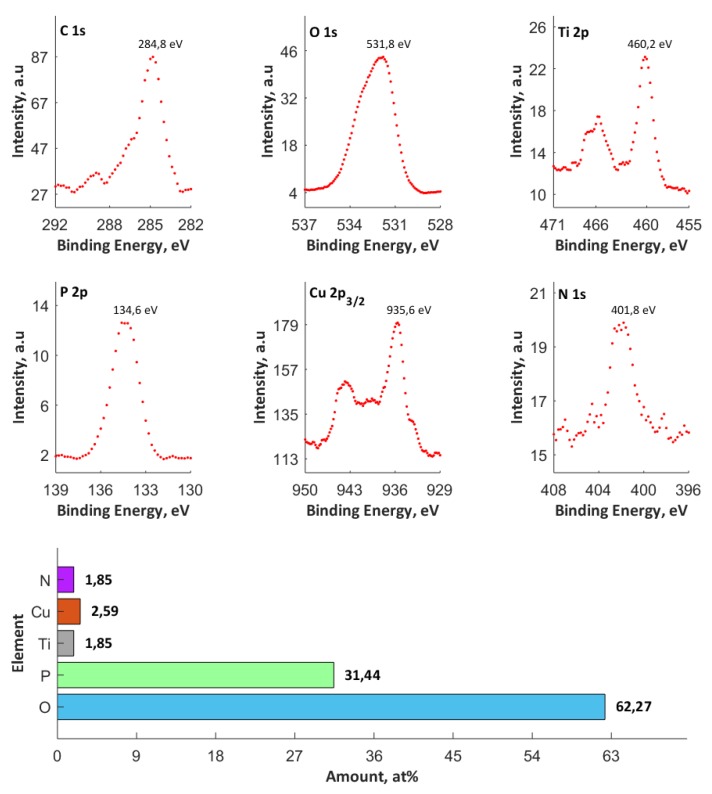
XPS quantitative analysis of the chemical composition of surface layers of an exemplary PEO coating containing maximum amount of copper inside (650 g/dm^3^ | 650 V).

**Table 1:** Examples of water electrolytes containing phosphates and poly-phosphates compounds used in plasma electrolytic oxidation processes.

Electrolytes	Ref.
(NaPO_3_)_6_, NaF, NaAlO_2_	[12]
(NaPO_3_)_6_, CaO, Na_2_H_2_EDTA, KOH	[13]
(NaPO_3_)_6,_ Na_2_SiO_3_, NaAlO_2_	[14]
Na_4_P_2_O_7_, KOH, NaAlO_2_	[15]
Na_3_PO_4_, (NH_4_)_2_HPO_4_, C_2_H_7_NO_2_, KOH, Na_2_SO_4_, (HOCH_2_)_3_CNH_2_	[16]
Na_3_PO_4_, Na_2_B_4_O_7_, Na_3_WO_4_	[17]
Na_3_PO_4_, α-Al_2_O_3_	[18]
Na_3_PO_4_, Na_2_SiO_3_	[14]
Na_3_PO_4_, FeSO_4_, Co(CH_3_COO)_2_, Ni(CH_3_COO)_2_, K_2_ZrF_6_	[19]
Na_3_PO_4_, FeSO_4_	[20]
Na_3_PO_4_, Co(CH_3_COO)_2_	[21]
Na_3_PO_4_, NaAlO_2_, KOH, NaCl	[22]
NaH_2_PO_4_, (CH_3_COO)_2_Ca	[23]
Ca(H_2_PO_4_)_2_, CaO, Na_2_(EDTA), Na_2_SiO_3_	[24]

EDTA - ethylenediaminetetraacetic acid.

## Supplementary Materials

The following are available online at https://www.mdpi.com/1996-1944/13/6/1295/s1, Figure S1: Juxtaposition of exemplary SEM images (a–l) and 3D maps obtained by using CLSM technique (m–o) on the PEO coatings fabricated with an electrolyte of concentrated orthophosphoric acid at voltages: 450 V (a, d, g, j, m), 550 V (b, e, h, k, n), and 650 V (c, f, i, l), Figure S2: Examples of SEM images (a–l) and 3D maps obtained by CLSM (m–o) on the PEO coatings fabricated in an electrolyte consisting of orthophosphoric acid and copper (II) nitrate (V) trihydrate of concentration 10 g/dm^3^ at the voltages: 450 V (a, d, g, j, m), 550 V (b, e, h, k, n), and 650 V (c, f, i, l), Figure S3: Examples of SEM SEM images (a–l) and 3D maps obtained by using CLSM technique (m–o) on the PEO coatings fabricated in the electrolyte consisting of the orthophosphoric acid and the copper (II) nitrate (V) trihydrate of concentration 50 g/dm^3^ at the voltages: 450 V (a, d, g, j, m), 550 V (b, e, h, k, n), and 650 V (c, f, i, l), Figure S4: Examples of SEM images (a–l) and 3D maps obtained by CLSM (m–o) on the PEO coatings fabricated in an electrolyte consisting of orthophosphoric acid and copper (II) nitrate (V) trihydrate of concentration 200 g/dm^3^ at the voltages: 450 V (a, d, g, j, m), 550 V (b, e, h, k, n), and 650 V (c, f, i, l), Figure S5: Examples of SEM images (a–l) and 3D maps obtained by CLSM (m–o) on the PEO coatings fabricated in an electrolyte consisting of orthophosphoric acid and copper (II) nitrate (V) trihydrate of concentration 350 g/dm^3^ at voltages: 450 V (a, d, g, j, m), 550 V (b, e, h, k, n), and 650 V (c, f, i, l), Figure S6: Examples of SEM images (a–l) and 3D maps obtained by CLSM (m–o) on the PEO coatings fabricated in an electrolyte consisting of orthophosphoric acid and copper (II) nitrate (V) trihydrate of concentration 500 g/dm^3^ at the voltages: 450 V (a,d,g,j,m), 550 V (b, e, h, k, n), and 650 V (c, f, i, l), Figure S7: Examples of SEM images (a–l) and 3D maps obtained by CLSM (m–o) on the PEO coatings fabricated in an electrolyte consisting of orthophosphoric acid and copper (II) nitrate (V) trihydrate of concentration 650 g/dm^3^ at the voltages: 450 V (a, d, g, j, m), 550 V (b, e, h, k, n), and 650 V (c, f, i, l)., Figure S8: XPS spectra obtained for the PEO coating fabricated in the electrolyte consisting of orthophosphoric acid and copper(II) nitrate(V) trihydrate at concentration 50 g/dm^3^ with a voltage of 550 V, Figure S9: XPS spectra obtained for the PEO coating fabricated in the electrolyte consisting of orthophosphoric acid and copper(II) nitrate(V) trihydrate at concentration 350 g/dm^3^ and voltage of 550 V, Figure S10: XPS spectra obtained for the PEO coating fabricated in the electrolyte consisting of orthophosphoric acid and copper(II) nitrate(V) trihydrate at concentration 500 g/dm^3^ and voltage of 450 V, Figure S11: XPS spectra obtained for the PEO coating fabricated in the electrolyte consisting of orthophosphoric acid and copper(II) nitrate(V) trihydrate at concentration 500 g/dm^3^ and voltage of 500 V, Figure S12: XPS spectra obtained for the PEO coating fabricated in the electrolyte consisting of orthophosphoric acid and copper(II) nitrate(V) trihydrate at concentration 500 g/dm^3^ and voltage of 550 V, Figure S13: XPS spectra obtained for the PEO coating fabricated in the electrolyte consisting of orthophosphoric acid and copper(II) nitrate(V) trihydrate at concentration 500 g/dm^3^ and voltage of 600 V, Figure S14: XPS spectra obtained for the PEO coating fabricated in the electrolyte consisting of orthophosphoric acid and copper(II) nitrate(V) trihydrate at concentration 500 g/dm^3^ and voltage of 650 V, Figure S15: XPS spectra obtained for the PEO coating fabricated in the electrolyte consisting orthophosphoric acid and the copper(II) nitrate(V) trihydrate of concentration 650 g/dm^3^ and voltage of 450 V, Figure S16: XPS spectra obtained for the PEO coating fabricated in the electrolyte consisting orthophosphoric acid and the copper(II) nitrate(V) trihydrate of concentration 650 g/dm^3^ and voltage of 650 V, Figure S17: XRD diffractogram obtained for the PEO coating fabricated in the electrolyte consisting of orthophosphoric acid and copper(II) nitrate(V) trihydrate at concentration 10 g/dm^3^ with a voltage of 550 V, Figure S18: XRD diffractogram obtained for the PEO coating fabricated in the electrolyte consisting of orthophosphoric acid and copper(II) nitrate(V) trihydrate at concentration 50 g/dm^3^ and voltage of 550 V, Figure S19: XRD diffractogram obtained for the PEO coating fabricated in the electrolyte consisting of orthophosphoric acid and copper(II) nitrate(V) trihydrate at concentration 200 g/dm^3^ and voltage of 550 V, Figure S20: XRD diffractogram obtained for the PEO coating fabricated in the electrolyte consisting of orthophosphoric acid and copper(II) nitrate(V) trihydrate at concentration 350 g/dm^3^ and voltage of 550 V, Figure S21: XRD diffractogram obtained for the PEO coating fabricated in the electrolyte consisting of orthophosphoric acid and copper(II) nitrate(V) trihydrate at concentration 500 g/dm^3^ and voltage of 550 V, Figure S22: XRD diffractogram obtained for the PEO coating fabricated in the electrolyte consisting of orthophosphoric acid and copper(II) nitrate(V) trihydrate at concentration 500 g/dm^3^ and voltage of 450 V, Figure S23: XRD diffractogram obtained for the PEO coating fabricated in the electrolyte consisting of orthophosphoric acid and copper(II) nitrate(V) trihydrate at concentration 500 g/dm^3^ and voltage of 650 V, Figure S24: XRD diffractogram obtained for the PEO coating fabricated in the electrolyte consisting orthophosphoric acid and the copper(II) nitrate(V) trihydrate of concentration 650 g/dm^3^ and voltage of 550 V, Table S1: Results of surface topography parameters using CLSM method, on a titanium sample after PEO processing in a pure concentrated orthophosphoric acid electrolyte at different voltages 450 V, 550 V, and 650 V, Table S2: Results of surface topography parameters for a titanium sample after PEO processing in an electrolyte with 10 g/dm^3^ of Cu(NO_3_)_2_∙3H_2_O amounting at the voltages of 450 V, 550 V, and 650 V, Table S3: Results of surface topography parameters for a titanium sample after PEO processing in an electrolyte with 50 g/dm^3^ of Cu(NO_3_)_2_∙3H_2_O amounting at the voltages of 450 V, 550 V, and 650 V, Table S4: Results of surface topography parameters for a titanium sample after PEO processing in an electrolyte with 200 g/dm^3^ of Cu(NO_3_)_2_∙3H_2_O amounting at the voltages of 450 V, 550 V, and 650 V, Table S5: Results of surface topography parameters for a titanium sample after PEO processing in an electrolyte with 350 g/dm^3^ of Cu(NO_3_)_2_∙3H_2_O amounting at the voltages of 450 V, 550 V, and 650 V, Table S6: Results of surface topography parameters for a titanium sample after PEO processing in an electrolyte with 500 g/dm^3^ of Cu(NO_3_)_2_∙3H_2_O amounting at the voltages of 450 V, 550 V, and 650 V, Table S7: Results of surface topography parameters for a titanium sample after PEO processing in an electrolyte with 650 g/dm^3^ of Cu(NO_3_)_2_∙3H_2_O amounting at the voltages of 450 V, 550 V, and 650 V.
