# Supplementary material for: Phosphate Coatings Enriched with Copper on Titanium Substrate Fabricated Via DC-PEO Process
Source: Materials (Basel). 2020 Mar 13;13(6):1295. doi: 10.3390/ma13061295 (PMC7142448; doi:10.3390/ma13061295)
Supplement: Supplementary file 1 [file materials-13-01295-s001.pdf]

materials

Supplement

Phosphate Coatings Enriched with Copper on Titanium Substrate Fabricated Via DC-PEO Process

Krzysztof Rokosz [1*], Tadeusz Hryniewicz [1], Wojciech Kacalak[1], Katarzyna Tandecka[1], Steinar Raaen[2], Sofia Gaiaschi [3], Patrick Chapon [3], Winfried Malorny [4], Dalibor Matýsek [5], Kornel Pietrzak[1], and Łukasz Dudek [1]

[1] Department of Engineering and Informatics Systems, Faculty of Mechanical Engineering, Koszalin University of Technology, Koszalin 75620, Poland; rokosz@tu.koszalin.pl (K.R.); Tadeusz.Hryniewicz@tu.koszalin.pl (T.H.); Wojciech.Kacalak@tu.koszalin.pl (W.K); Katarzyna.Tandecka@tu.koszalin.pl (K.T.); kornel.pietrzak@s.tu.koszalin.pl (K.P.); lukasz.dudek@tu.koszalin.pl (Ł.D.)

[2] Department of Physics, Norwegian University of Science and Technology (NTNU), Trondheim 7491, Norway; steinar.raaen@ntnu.no

[3] HORIBA FRANCE S.A.S., Boulevard Thomas Gobert - Passage Jobin Yvon, Palaiseau 91120, France; sofia.gaiaschi@horiba.com (S.G.); patrick.chapon@horiba.com (P.C.)

[4] Hochschule Wismar-University of Applied Sciences Technology, Business and Design, Faculty of Engineering, Wismar 23966, Germany; winfried.malorny@hs-wismar.de

[5] Institute of Geological Engineering, Faculty of Mining and Geology, VŠB—Technical University of Ostrava, Ostrava 70833, Czech Republic, dalibor.matysek@vsb.cz

* Correspondence: rokosz@tu.koszalin.pl; Tel.: +48-94-3478354

Received: 7 February 2020; Accepted: 10 March 2020; Published: date

1. Characterization Methods of PEO Coatings

Images of the obtained coatings were completed by means of the scanning electron microscope FEI Quanta 250 that was equipped with a field emission electron gun. Each time the SEM imaging was carried out under a high vacuum, employing Everhart–Thornley detector under the secondary electron imaging mode.

Analysis of the atomic composition of the obtained coatings was performed by means of the energy-dispersive X-ray spectroscopy (EDS), while using the ultra-dry EDS detector of Thermo Scientific Co., co-operating with a scanning electron microscope FEI Quanta 250. For a quantitative determination, the atomic contents of Ti (Kα), P (Kα), and Cu (Kα) were taken into account. The studies were carried out on 10 samples, at the magnification of 500×, while using the accelerating voltage of 20 kV.

The XPS studies were performed by means of the SCIENCE SES 2002 apparatus, taking advantage of a monochromatic source of the X-ray radiation Al Kα ($h\nu$ = 1486.6 eV), which was operating at the current of 18.7 mA and the voltage of 13.02 kV. Each time the analyzed surface area of the sample was 3 mm^2, the pass energy of 500 eV with the leap of 0.2 eV and the leap time equaling 200 ms. The device power supply was stabilized to increase the energy accuracy that was supplied to the source of radiation. Measurements were performed in a vacuum chamber with an ultra-high vacuum equaling 6×10^{-8} Pa.

For each of the studied samples, the XPS analysis for the binding energies of titanium (Ti 2p), phosphorus (P 2p), copper (Cu 2p3/2), oxygen (O 1s), carbon (C 1s), and nitrogen (N 1s), were done. All of the presented herewith XPS spectra have been calibrated against the binding energies of C–C, C–H for carbon (C 1s), equaling 284.8 eV, and the analyses of the obtained data were carried out while using the softwares of Casa XPS 2.3.14 (Casa Software Ltd., Teignmouth, Devon, UK).

The XRD studies were carried out in the porous PEO coatings. The diffractograms that were presented in this work were obtained by means of the D8 Advance measuring system of Bruker's Co. (BRUKER Corporation, Billerica, MA, USA), equipped with a focusing

circle/circumference//district of Bragg-Brentano type (2Θ/Θ), the source of X-ray radiation Cu K*α* (40 kV, 40 mA), and the 192-channel detector LynxEye with a nickel aperture/diaphragm/cover.

The studies of the surface topography were carried out by using a confocal laser microscope LEXT OLS 4000 by Olympus Co., which was equipped with the object lens/objective LEXT OLS 4000 by Olympus Co. (aperture 0.95, operating distance 0.35 mm). In the laser mode, a semiconductive source of radiation for the wave of 405 nm, operating on a permanent basis of a maximum power of 6 mW, and the divergency in the range from 0.12 up to 0.41 rad, was used. The white light LED was used in the optical mode. A photomultiplier was responsible for the detection system in a laser mode and a single panel CCD (2×10^8 pixels) – for a laser mode.

The profile analysis of the depth distribution of the elements of the obtained PEO coatings was performed by means of the GDOES apparatus. For this purpose, a Horiba Scientific Profiler 2 was used by taking advantage of the asynchronous frequency pulse generator of plasma. The studies were carried out in a low pressure (700 Pa) chamber, the operational frequency of the plasma generator was equal 3000 Hz, under the discontinuous mode equaling 0.25 cycle of the power of 40 W in the argon environment by using the anode with the diameter of 4 mm.

2. SEM and CLSM Results

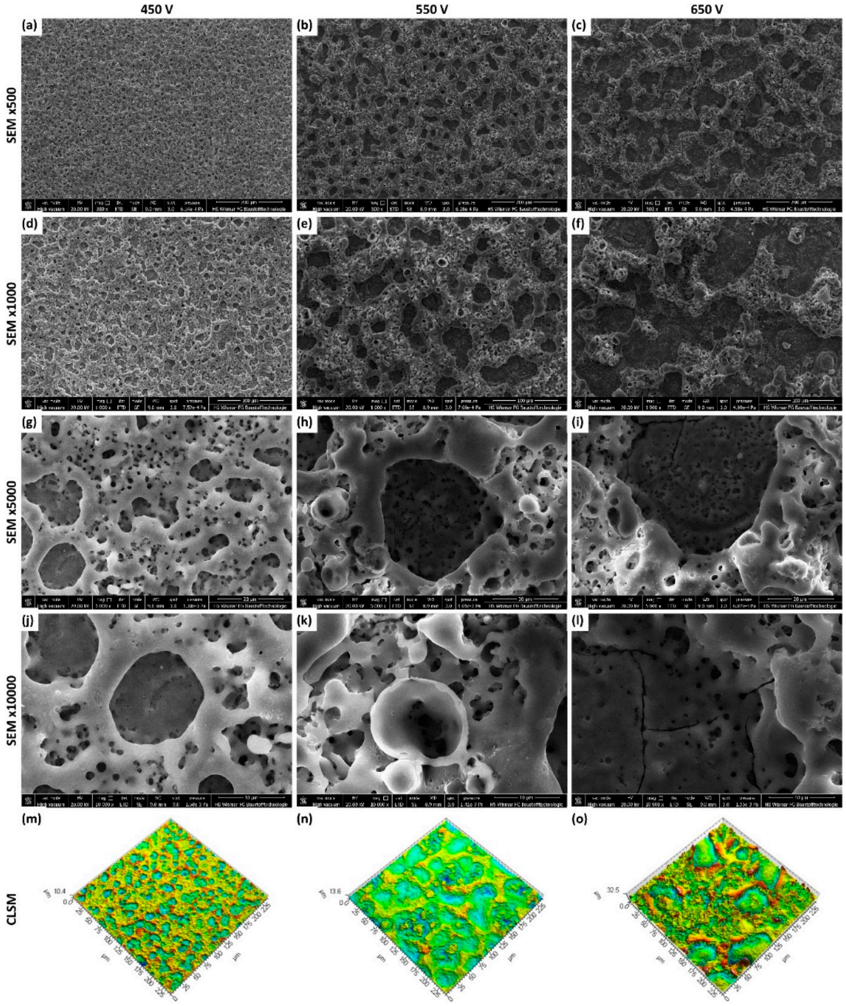

Figure S1. Juxtaposition of exemplary SEM images (**a–l**) and three-dimensional (3D) maps obtained by using CLSM technique (**m–o**) on the PEO coatings fabricated with an electrolyte of concentrated orthophosphoric acid at voltages: 450 V (**a, d, g, j, m**), 550 V (**b, e, h, k, n**), and 650 V (**c, f, i, l**).

Table S1. Results of surface topography parameters using CLSM method, on a titanium sample after PEO processing in a pure concentrated orthophosphoric acid electrolyte at different voltages 450 V, 550 V, and 650 V.

**3D Roughness**	***Sp*, μm**			***Sv*, μm**			***Sz*, μm**			***Sa*, μm**		
**Voltage *U*, V**	**450**	**550**	**650**	**450**	**550**	**650**	**450**	**550**	**650**	**450**	**550**	**650**
Average	4.64	6.29	15.20	6.39	6.78	16.37	11.03	13.08	31.57	1.13	1.03	2.87
Std. deviation	0.24	1.21	0.60	0.87	0.53	0.94	0.64	1.08	1.44	0.01	0.01	0.23
Range	0.59	2.93	1.46	2.08	1.29	2.09	1.50	2.39	3.32	0.02	0.02	0.57

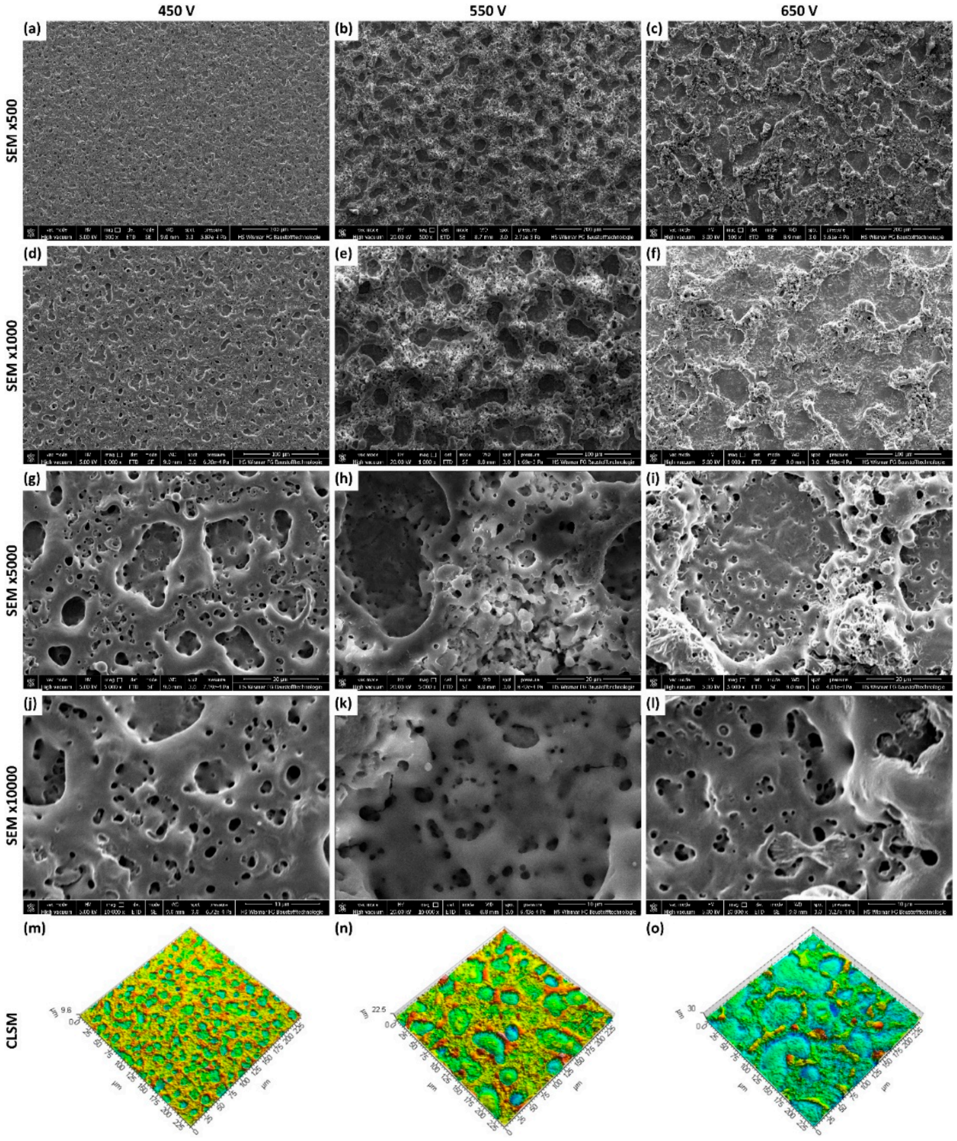

Figure S2. Examples of SEM images (**a–l**) and 3D maps obtained by CLSM (**m–o**) on the PEO coatings fabricated in an electrolyte consisting of orthophosphoric acid and copper (II) nitrate (V) trihydrate of concentration 10 g/dm^3 at the voltages: 450 V (**a, d, g, j, m**), 550 V (**b, e, h, k, n**), and 650 V (**c, f, i, l**).

Table S2. Results of surface topography parameters for a titanium sample after PEO processing in an electrolyte with 10 g/dm³ of $Cu(NO_3)_2{\cdot}3H_2O$ amounting at the voltages of 450 V, 550 V, and 650 V.

**3D Roughness**		***Sp*, μm**			***Sv*, μm**			***Sz*, μm**			***Sa*, μm**	
**Voltage *U*, V**	**450**	**550**	**650**	**450**	**550**	**650**	**450**	**550**	**650**	**450**	**550**	**650**
Average	6.24	12.57	16.77	5.51	12.37	12.98	11.75	24.94	29.75	1.00	2.35	2.37
Std. deviation	2.47	1.63	1.84	0.15	0.63	0.85	2.44	2.22	1.36	0.03	0.14	0.12
Range	5.42	3.98	4.38	0.34	1.39	1.81	5.49	5.36	3.13	0.07	0.31	0.30

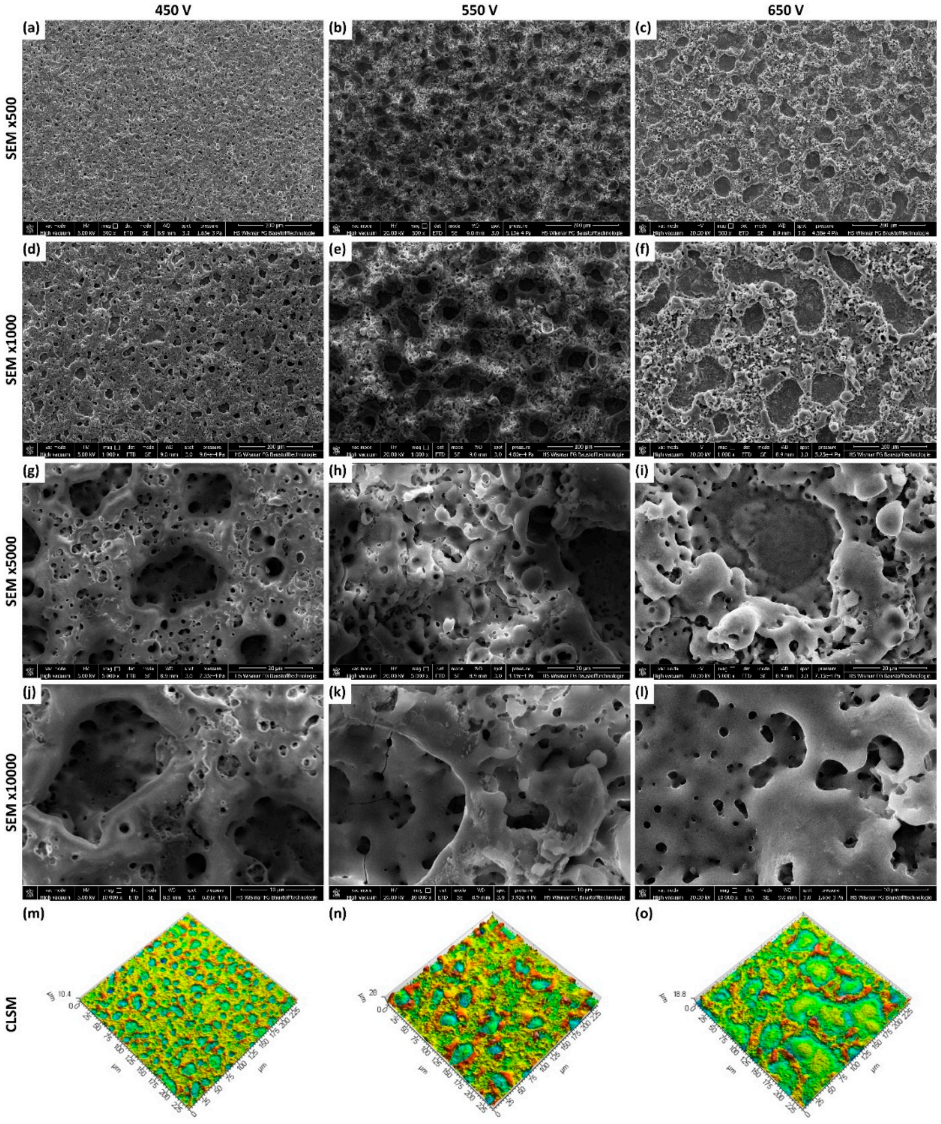

Figure S3. Examples of SEM SEM images (**a–l**) and 3D maps obtained by using CLSM technique (**m–o**) on the PEO coatings fabricated in the electrolyte consisting of the orthophosphoric acid and the copper (II) nitrate (V) trihydrate of concentration 50 g/dm³ at the voltages: 450 V (**a, d, g, j, m**), 550 V (**b, e, h, k, n**), and 650 V (**c, f, i, l**).

Table S3. Results of surface topography parameters for a titanium sample after PEO processing in an electrolyte with 50 g/dm^3 of $Cu(NO_3)_2{\cdot}3H_2O$ amounting at the voltages of 450 V, 550 V, and 650 V

**3D Roughness**	*Sp*, µm			*Sv*, µm			*Sz*, µm			*Sa*, µm		
**Voltage *U*, V**	**450**	**550**	**650**	**450**	**550**	**650**	**450**	**550**	**650**	**450**	**550**	**650**
Average	4.41	13.44	14.35	5.31	16.22	12.00	9.72	29.67	26.35	0.99	2.87	2.00
Std. deviation	0.44	0.42	0.97	0.22	0.54	0.87	0.50	0.94	1.45	0.06	0.07	0.12
Range	1.08	1.02	2.38	0.49	1.16	2.02	1.13	2.18	3.29	0.15	0.17	0.27

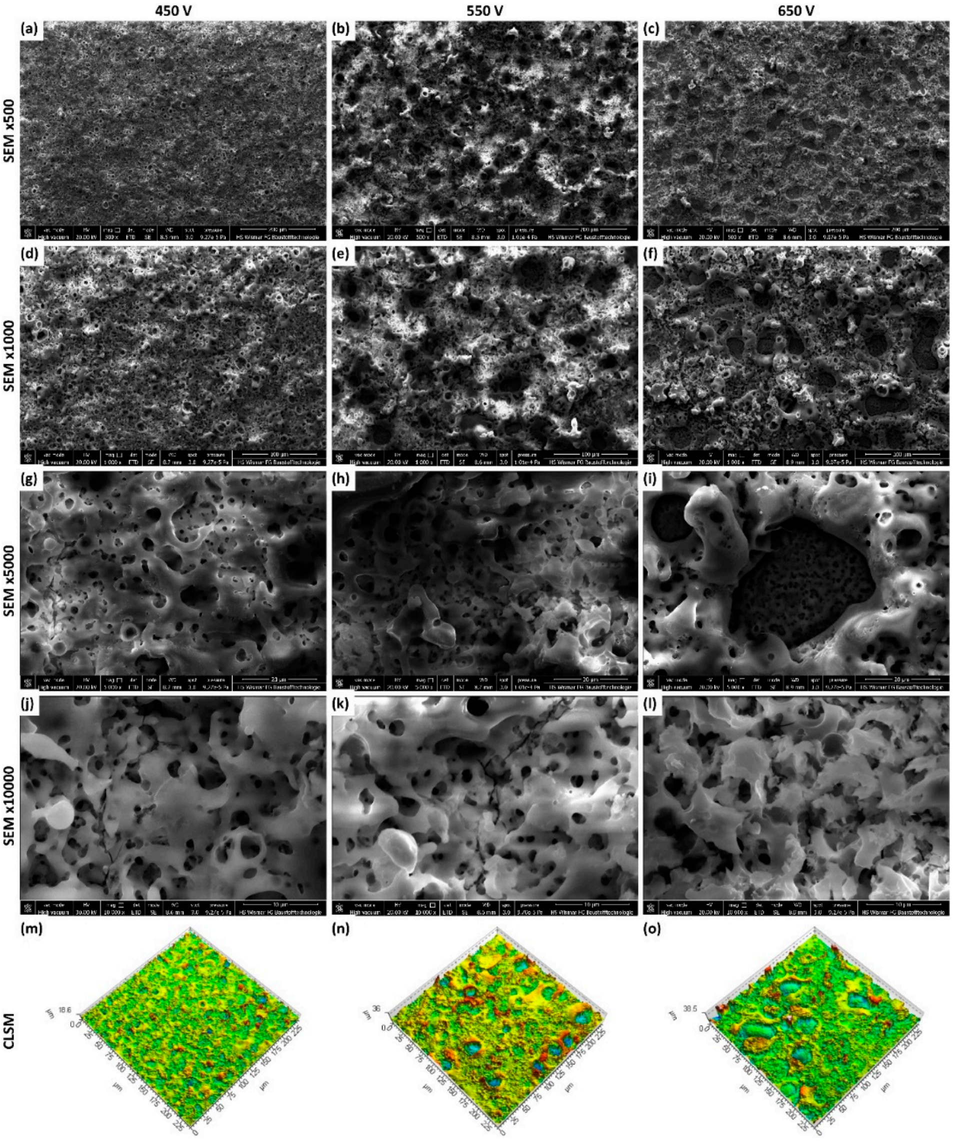

Figure S4. Examples of SEM images (**a–l**) and 3D maps obtained by CLSM (**m–o**) on the PEO coatings fabricated in an electrolyte consisting of orthophosphoric acid and copper (II) nitrate (V) trihydrate of concentration 200 g/dm^3 at the voltages: 450 V (**a, d, g, j, m**), 550 V (**b, e, h, k, n**), and 650 V (**c, f, i, l**).

Table S4. Results of surface topography parameters for a titanium sample after PEO processing in an electrolyte with 200 g/dm^3 of $Cu(NO_3)_2{\cdot}3H_2O$ amounting at the voltages of 450 V, 550 V, and 650 V.

**3D Roughness**	*Sp*, µm			*Sv*, µm			*Sz*, µm			*Sa*, µm		
**Voltage *U*, V**	**450**	**550**	**650**	**450**	**550**	**650**	**450**	**550**	**650**	**450**	**550**	**650**
Average	8.16	16.61	20.82	9.53	19.40	22.13	17.69	36.01	42.94	1.08	2.53	2.85
Std. deviation	0.73	1.78	2.34	0.45	0.82	2.42	0.80	0.99	3.42	0.06	0.09	0.32
Range	1.58	4.36	5.62	1.08	1.96	5.56	1.94	2.39	8.36	0.13	0.22	0.73

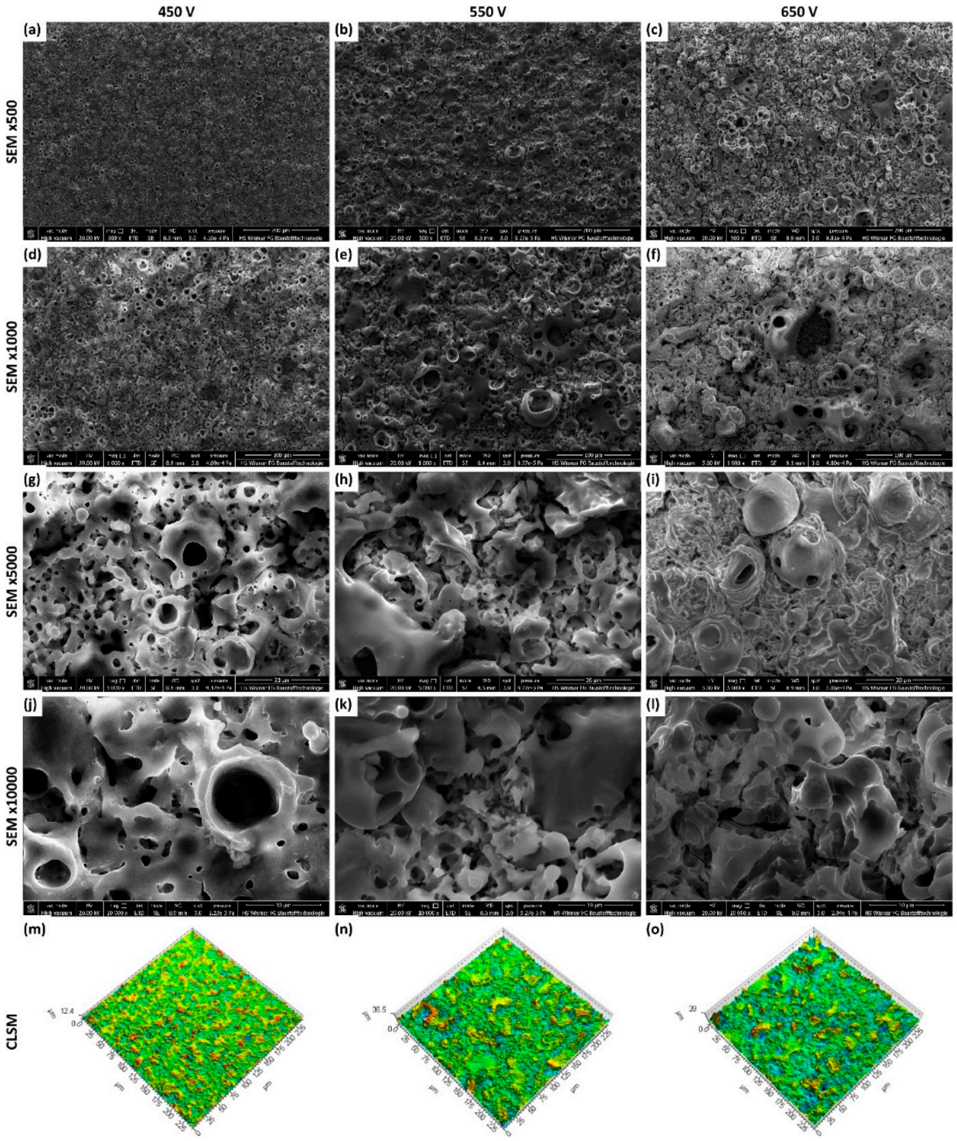

Figure S5. Examples of SEM images (**a–l**) and 3D maps that were obtained by CLSM (**m–o**) on the PEO coatings fabricated in an electrolyte consisting of orthophosphoric acid and copper (II) nitrate (V) trihydrate of concentration 350 g/dm^3 at voltages: 450 V (**a, d, g, j, m**), 550 V (**b, e, h, k, n**), and 650 V (**c, f, i, l**).

Table S5. Results of surface topography parameters for a titanium sample after PEO processing in an electrolyte with 350 g/dm^3 of $Cu(NO_3)_2{\cdot}3H_2O$ amounting at the voltages of 450 V, 550 V, and 650 V.

**3D Roughness**	***Sp*, μm**			***Sv*, μm**			***Sz*, μm**			***Sa*, μm**		
**Voltage *U*, V**	**450**	**550**	**650**	**450**	**550**	**650**	**450**	**550**	**650**	**450**	**550**	**650**
Average	7.42	16.74	19.75	6.90	19.10	18.35	14.31	35.84	38.10	0.98	2.48	2.87
Std. deviation	0.96	2.63	2.55	0.55	1.86	1.17	1.42	0.77	3.72	0.03	0.09	0.25
Range	2.32	5.60	6.21	1.19	4.01	2.78	3.46	1.71	9.00	0.08	0.20	0.62

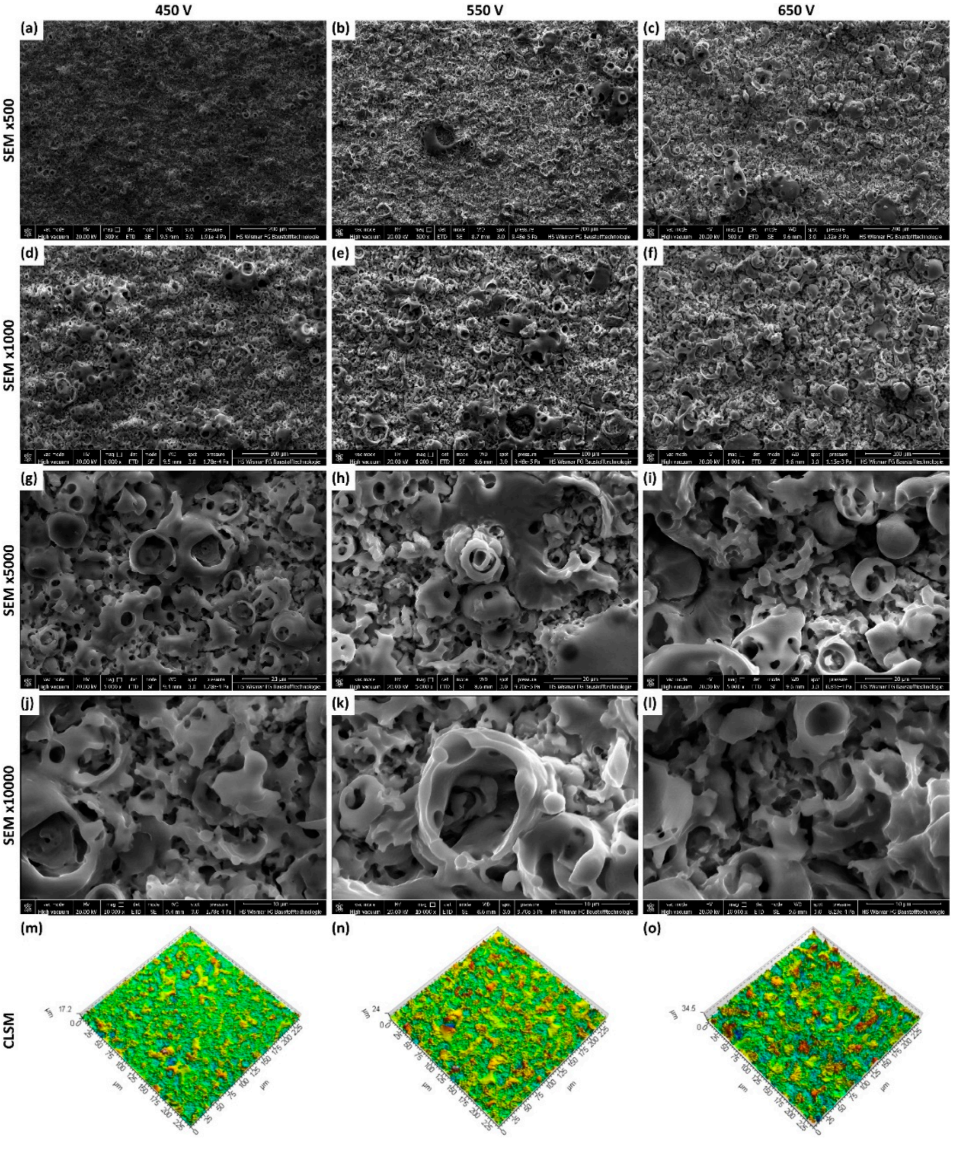

Figure S6. Examples of SEM images (**a–l**) and 3D maps obtained by CLSM (**m–o**) on the PEO coatings fabricated in an electrolyte consisting of orthophosphoric acid and copper (II) nitrate (V) trihydrate of concentration 500 g/dm^3 at the voltages: 450 V (**a,d,g,j,m**), 550 V (**b, e, h, k, n**), and 650 V (**c, f, i, l**).

Table S6. Results of surface topography parameters for a titanium sample after PEO processing in an electrolyte with 500 g/dm^3 of $Cu(NO_3)_2{\cdot}3H_2O$ amounting at the voltages of 450 V, 550 V, and 650 V.

3D Roughness	Sp, µm			Sv, µm			Sz, µm			Sa, µm		
**Voltage U, V**	**450**	**550**	**650**	**450**	**550**	**650**	**450**	**550**	**650**	**450**	**550**	**650**
Average	8.66	14.20	22.61	8.70	15.96	22.57	17.36	30.16	45.18	1.33	2.32	3.42
Std. deviation	0.65	1.43	4.38	0.61	3.16	5.04	0.86	4.49	9.11	0.03	0.26	0.10
Range	1.50	3.16	10.04	1.47	7.73	12.19	2.11	10.89	22.24	0.08	0.62	0.23

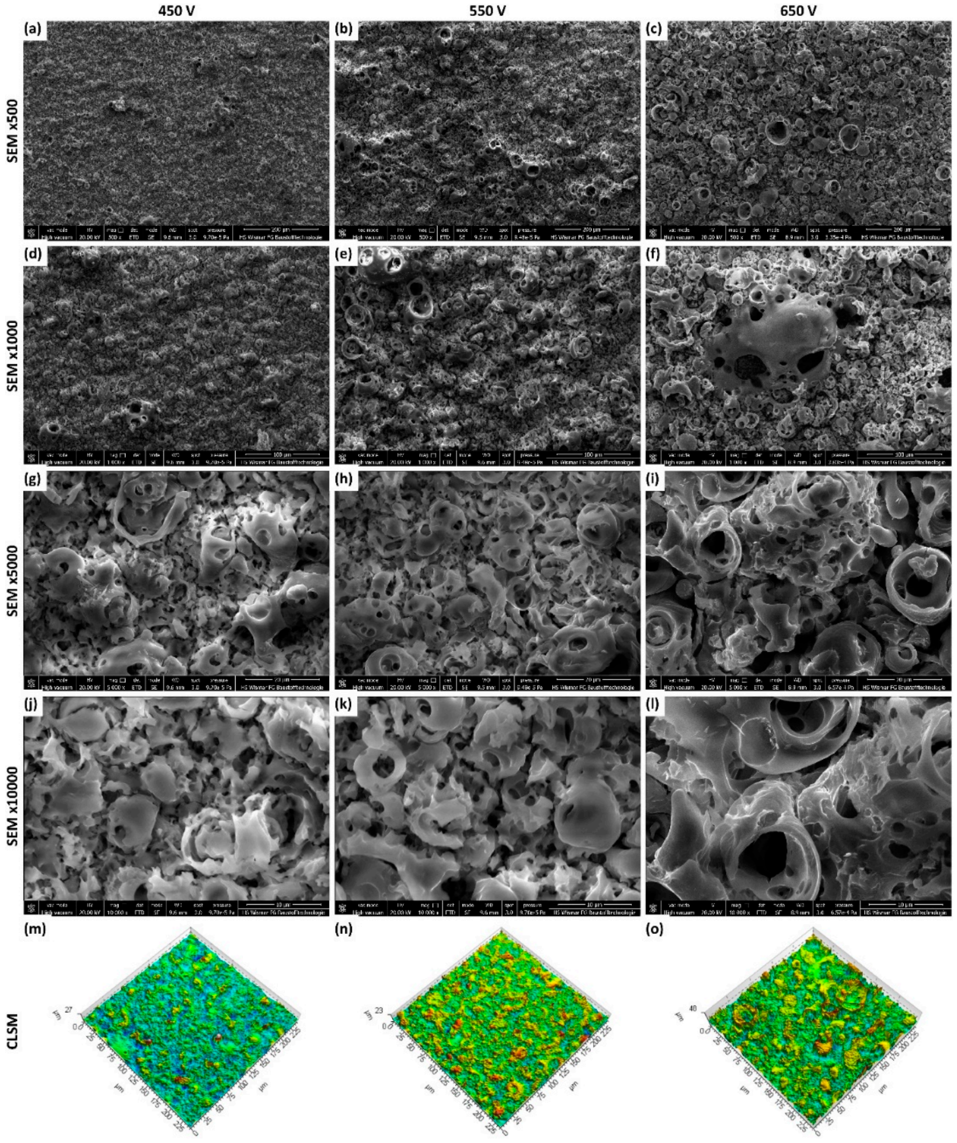

Figure S7. Examples of SEM images (**a–l**) and 3D maps obtained by CLSM (**m–o**) on the PEO coatings fabricated in an electrolyte consisting of orthophosphoric acid and copper (II) nitrate (V) trihydrate of concentration 650 g/dm^3 at the voltages: 450 V (**a, d, g, j, m**), 550 V (**b, e, h, k, n**), and 650 V (**c, f, i, l**).

Table S7. Results of surface topography parameters for a titanium sample after PEO processing in an electrolyte with 650 g/dm^3 of $Cu(NO_3)_2 \cdot 3H_2O$ amounting at the voltages of 450 V, 550 V, and 650 V

3D Roughness	*Sp*, µm			*Sv*, µm			*Sz*, µm			*Sa*, µm		
**Voltage *U*, V**	**450**	**550**	**650**	**450**	**550**	**650**	**450**	**550**	**650**	**450**	**550**	**650**
Average	15.58	12.74	20.58	11.87	13.53	18.86	27.45	26.27	39.44	2.06	2.09	3.59
Std. deviation	3.94	1.48	4.18	1.84	4.03	2.51	2.31	5.50	6.67	0.02	0.17	0.22
Range	9.33	3.27	9.88	4.11	8.98	6.11	5.66	12.24	15.99	0.04	0.39	0.52

3. XPS Results

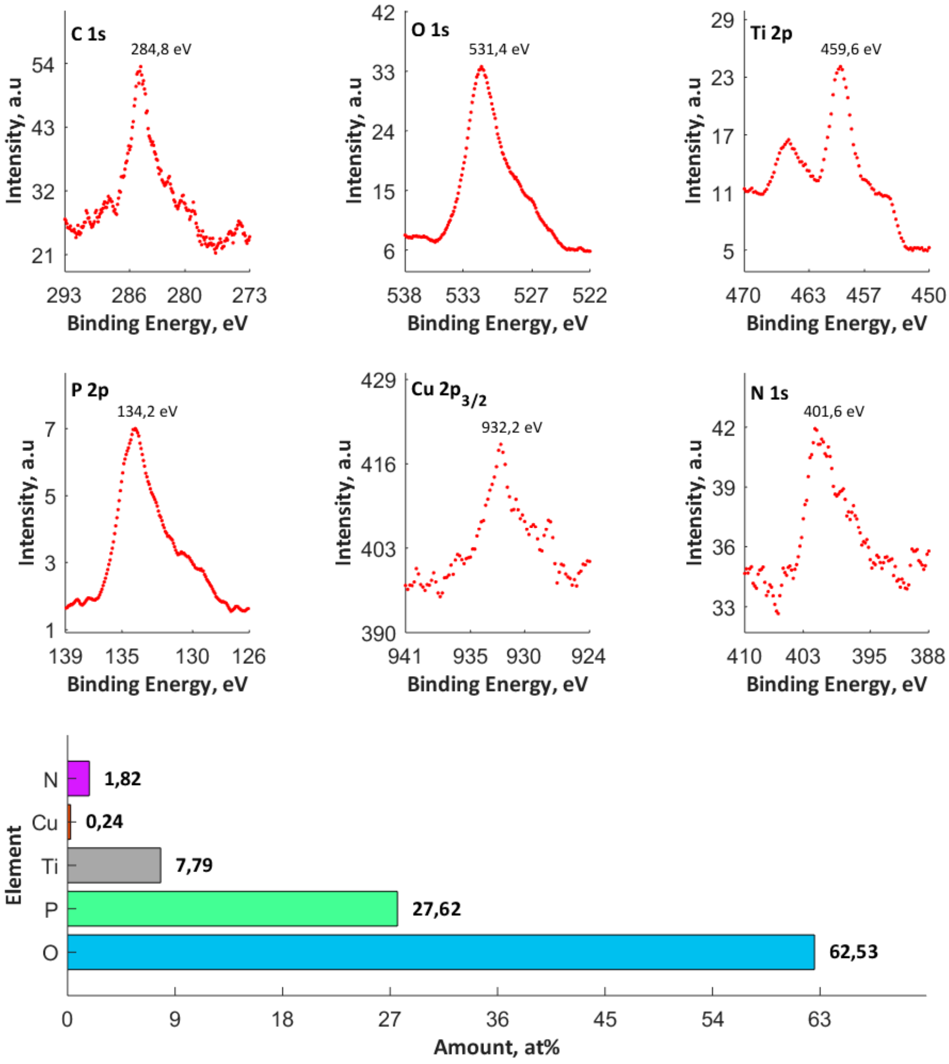

Figure S8. XPS spectra obtained for the PEO coating fabricated in the electrolyte consisting of orthophosphoric acid and copper(II) nitrate(V) trihydrate at concentration 50 g/dm^3 with a voltage of 550 V.

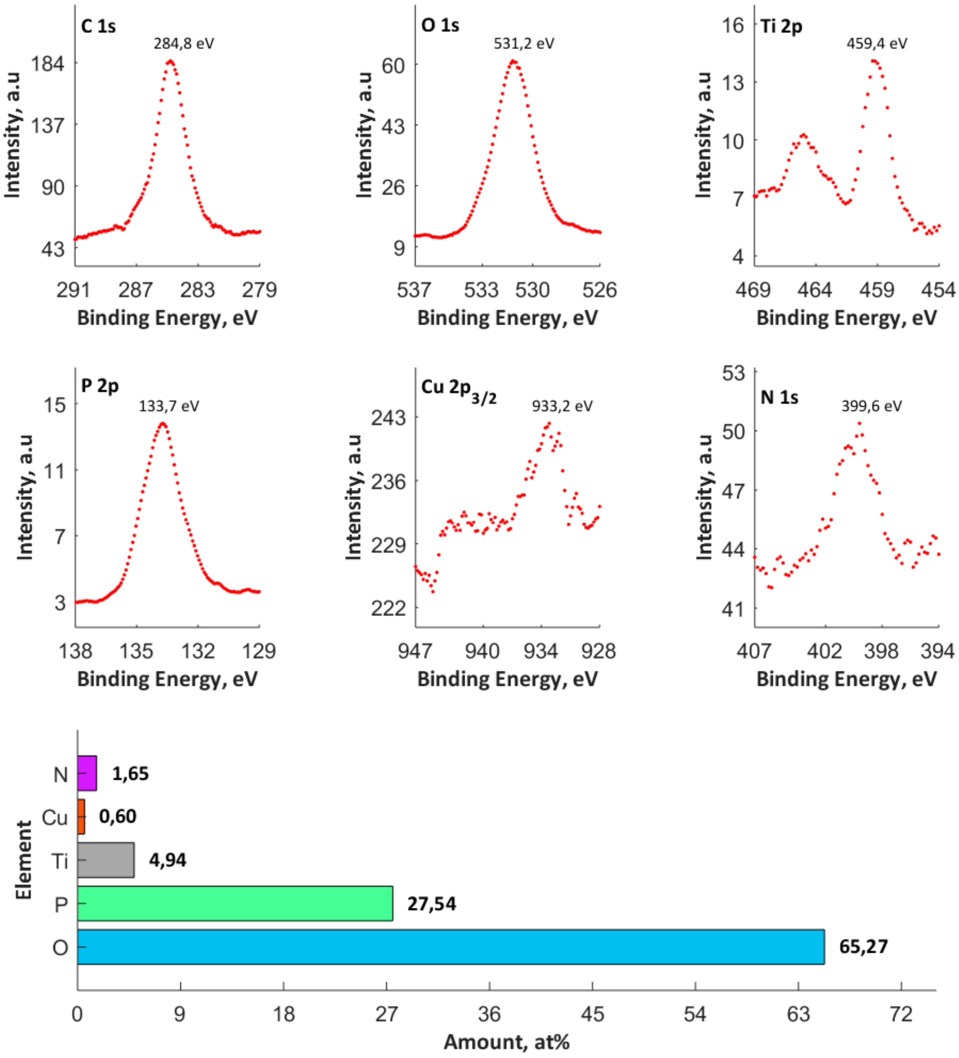

Figure S9. XPS spectra obtained for the PEO coating fabricated in the electrolyte consisting of orthophosphoric acid and copper(II) nitrate(V) trihydrate at concentration 350 g/dm^3 and voltage of 550 V.

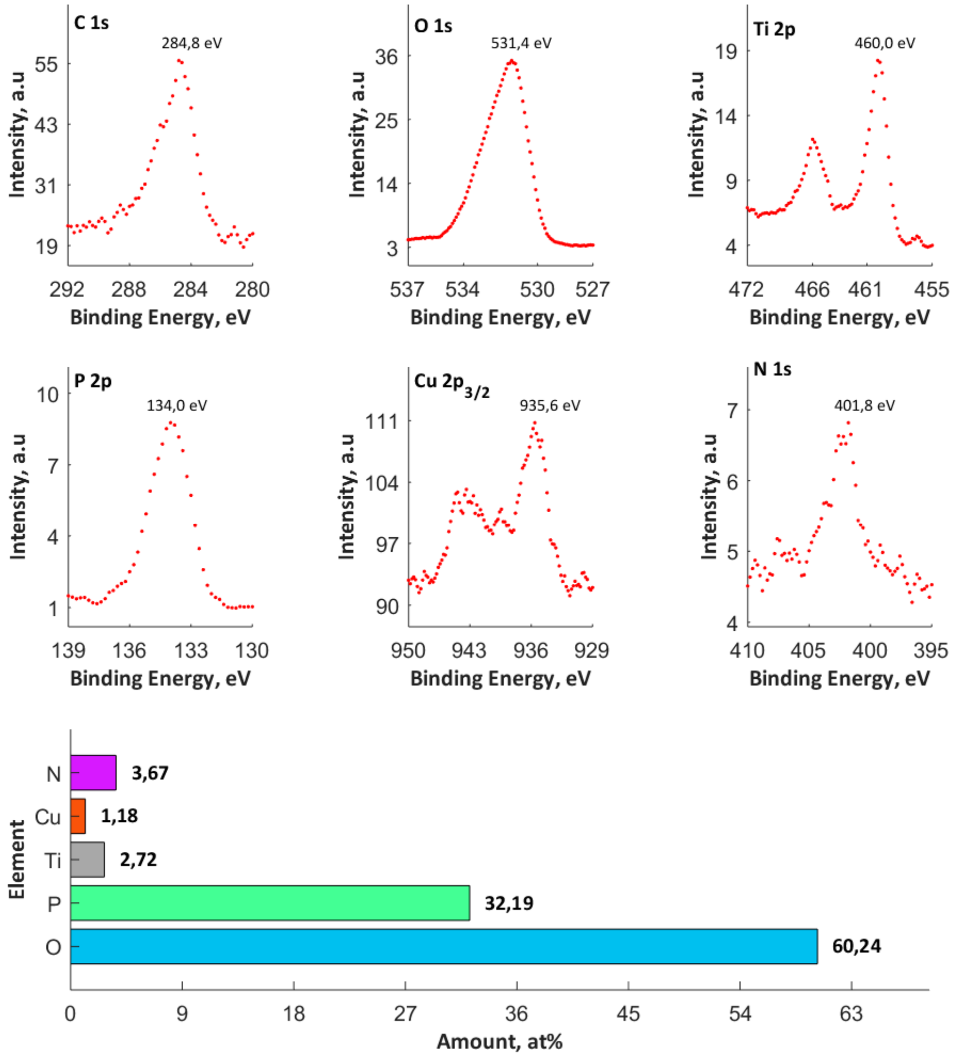

Figure S10. XPS spectra obtained for the PEO coating fabricated in the electrolyte consisting of orthophosphoric acid and copper(II) nitrate(V) trihydrate at concentration 500 g/dm^3 and voltage of 450 V.

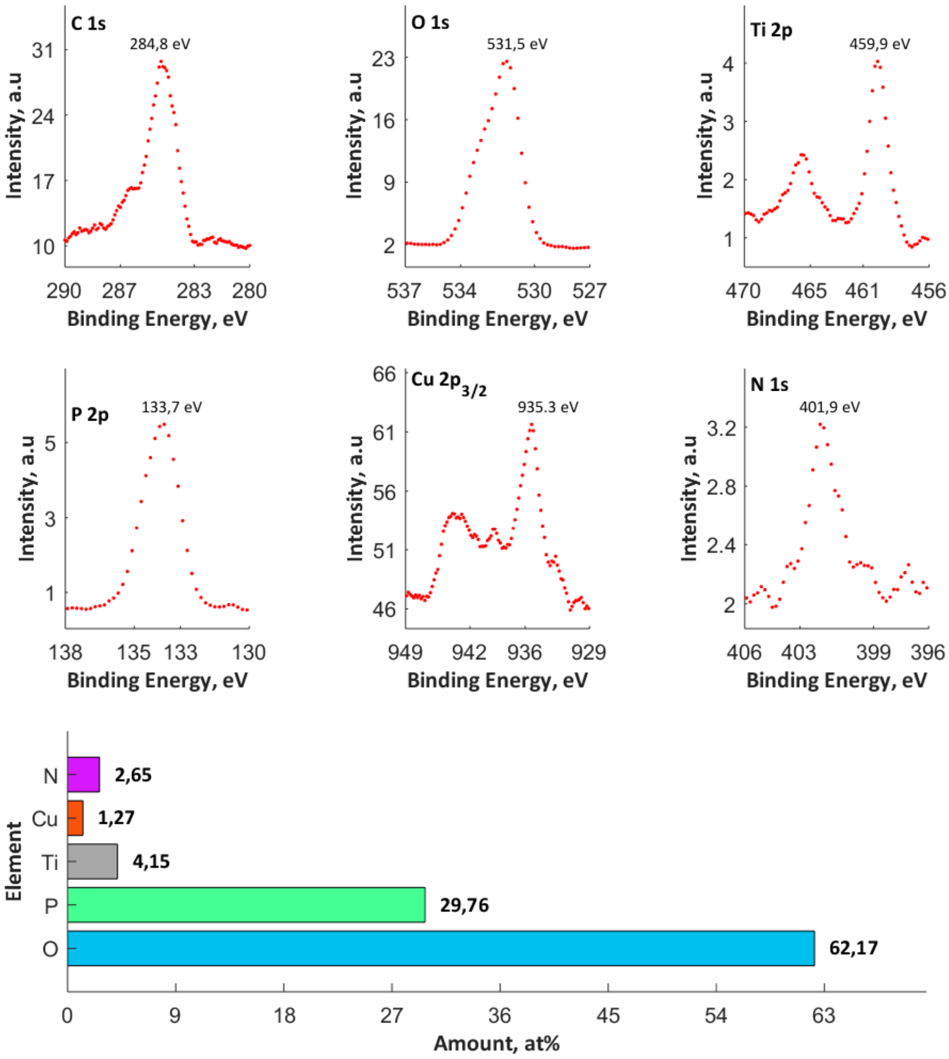

Figure S11. XPS spectra obtained for the PEO coating fabricated in the electrolyte consisting of orthophosphoric acid and copper(II) nitrate(V) trihydrate at concentration 500 g/dm^3 and voltage of 500 V.

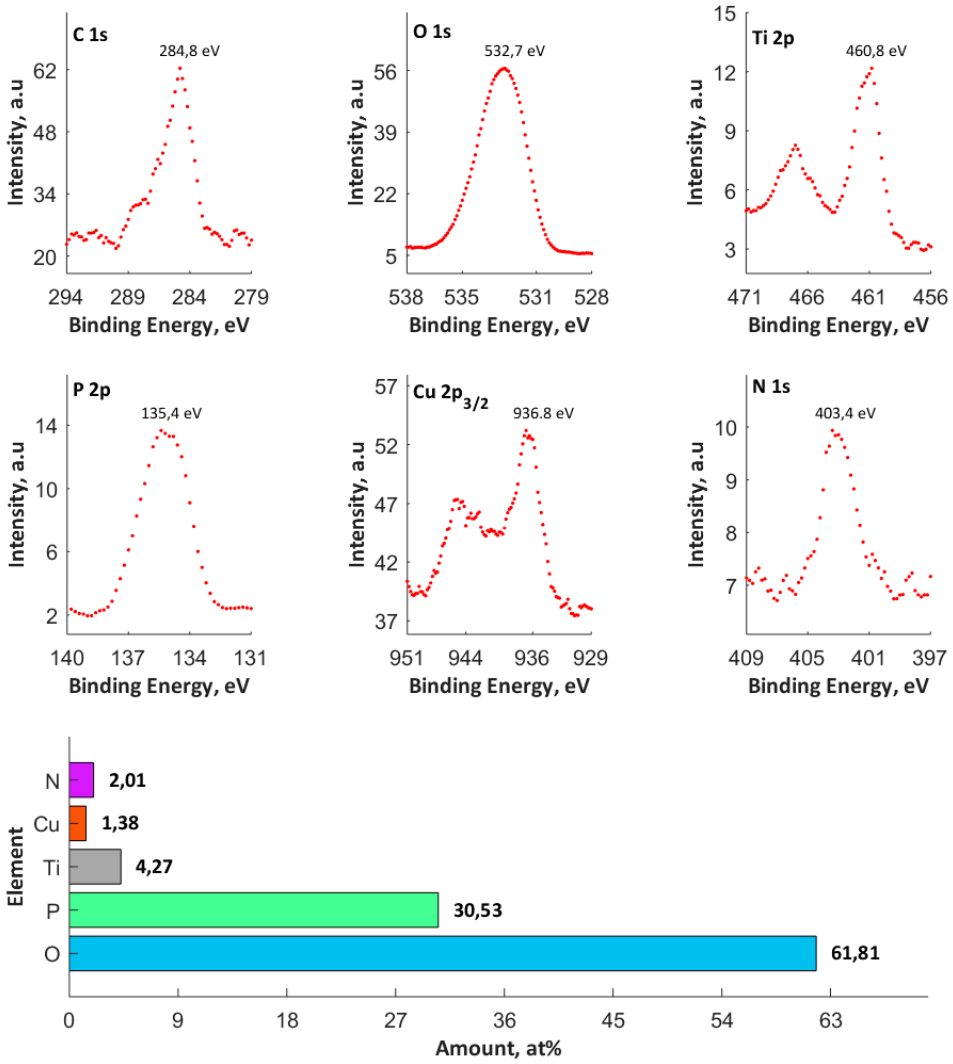

Figure S12. XPS spectra obtained for the PEO coating fabricated in the electrolyte consisting of orthophosphoric acid and copper(II) nitrate(V) trihydrate at concentration 500 g/dm^3 and voltage of 550 V.

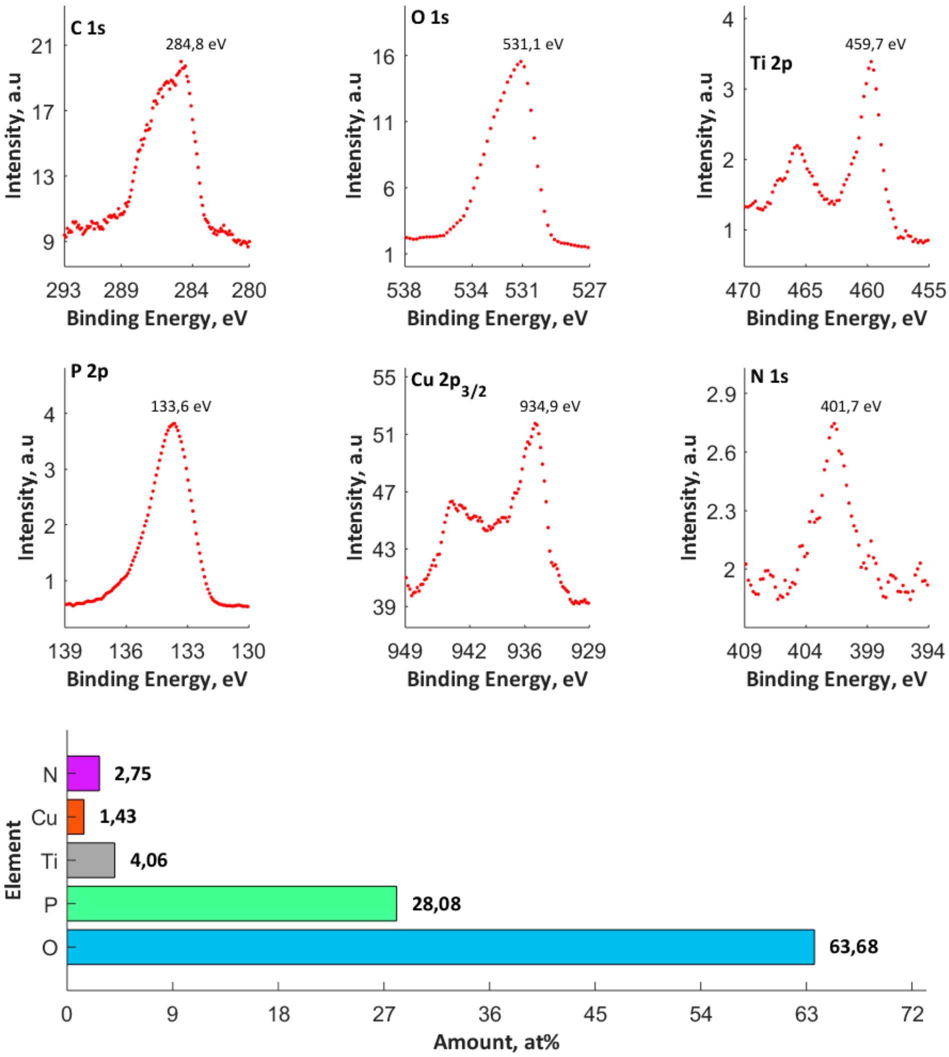

Figure S13. XPS spectra obtained for the PEO coating fabricated in the electrolyte consisting of orthophosphoric acid and copper(II) nitrate(V) trihydrate at concentration 500 g/dm^3 and voltage of 600 V.

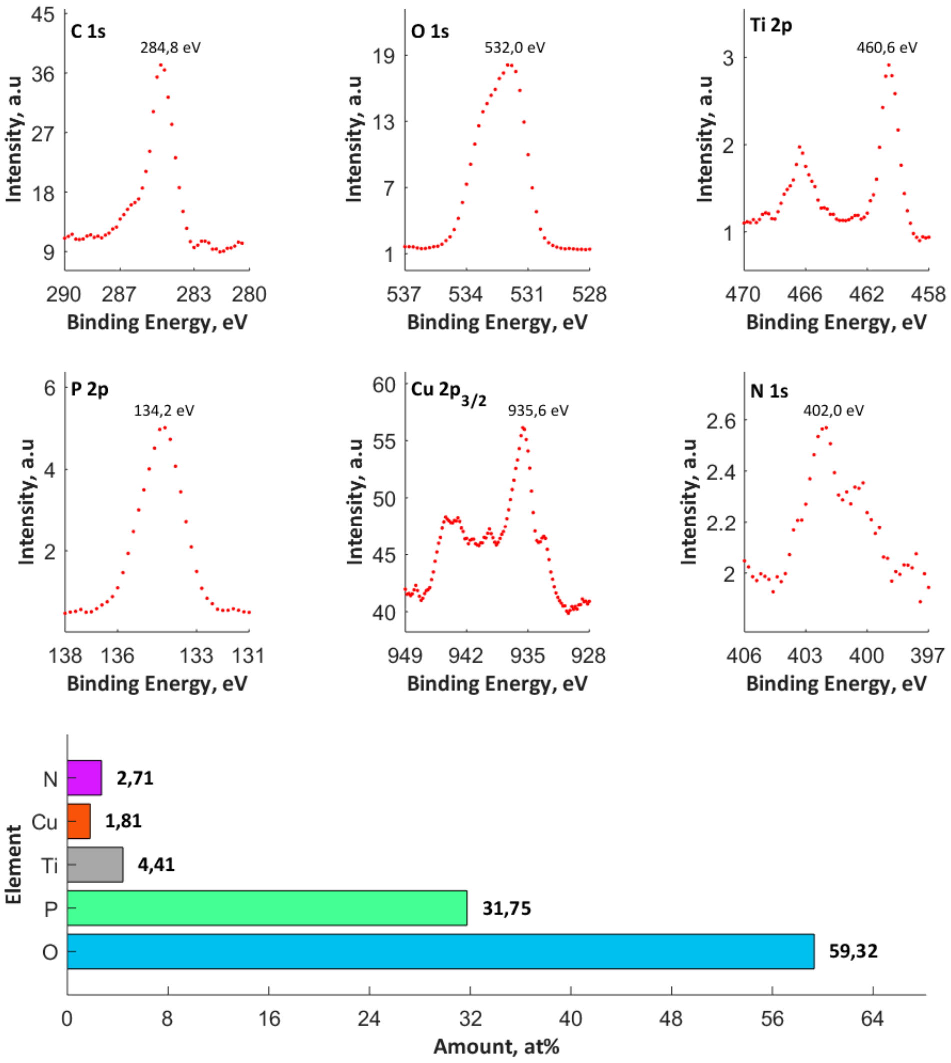

Figure S14. XPS spectra obtained for the PEO coating fabricated in the electrolyte consisting of orthophosphoric acid and copper(II) nitrate(V) trihydrate at concentration 500 g/dm^3 and voltage of 650 V.

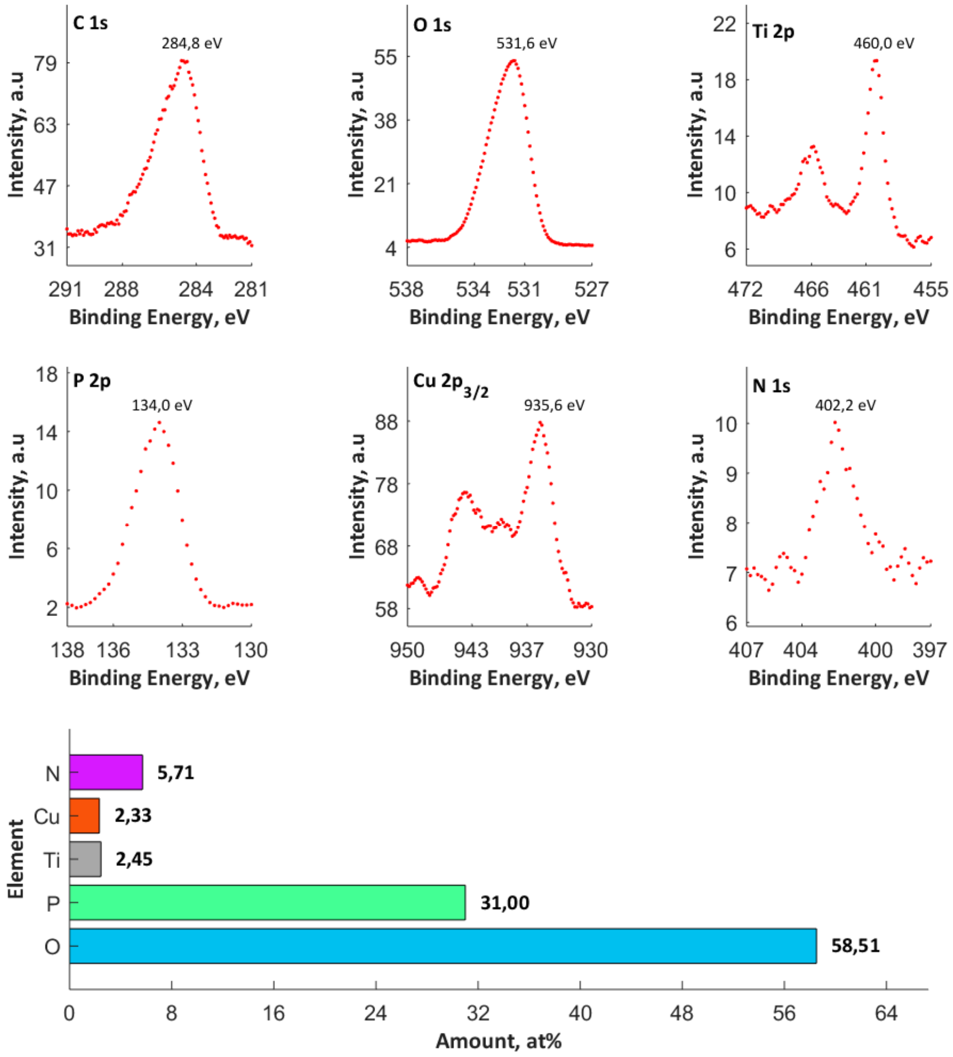

Figure S15. XPS spectra obtained for the PEO coating fabricated in the electrolyte consisting orthophosphoric acid and the copper(II) nitrate(V) trihydrate of concentration 650 g/dm^3 and voltage of 450 V.

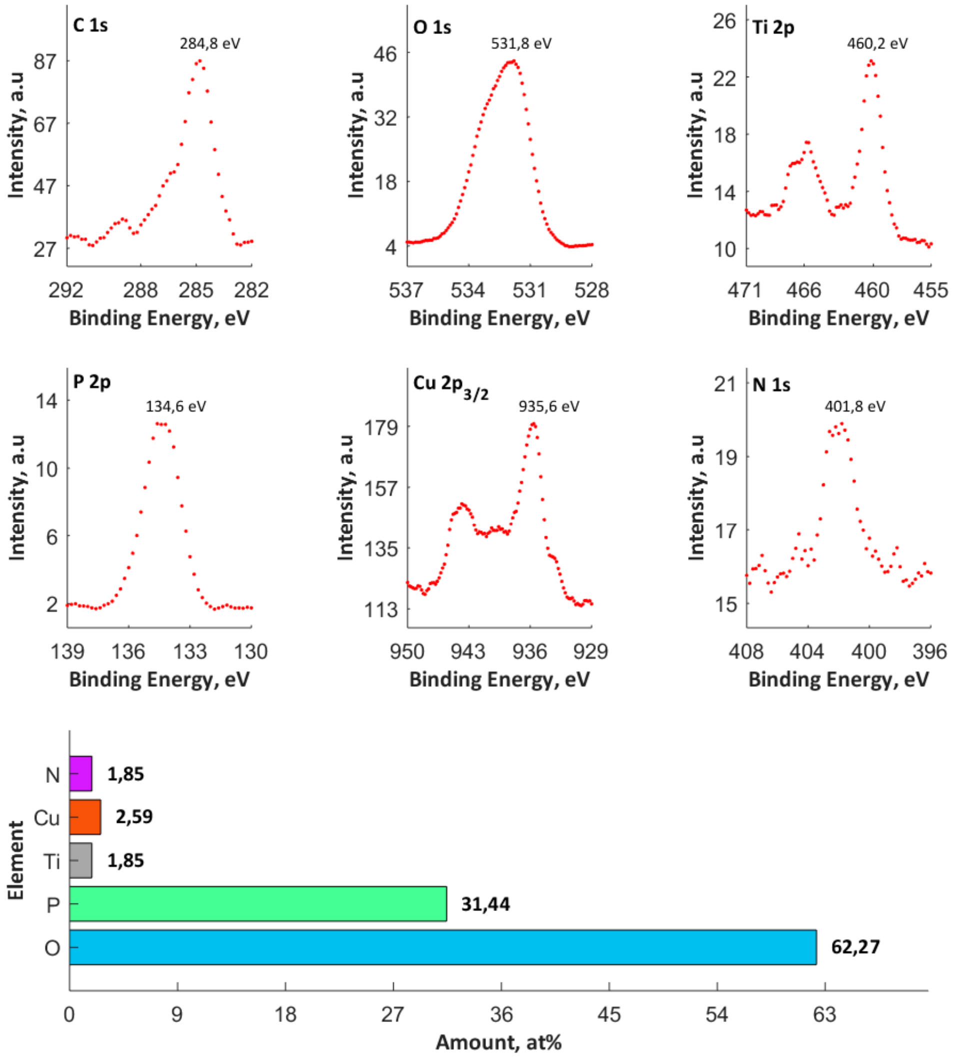

Figure S16. XPS spectra obtained for the PEO coating fabricated in the electrolyte consisting orthophosphoric acid and the copper(II) nitrate(V) trihydrate of concentration 650 g/dm³ and voltage of 650 V.

4. XRD results

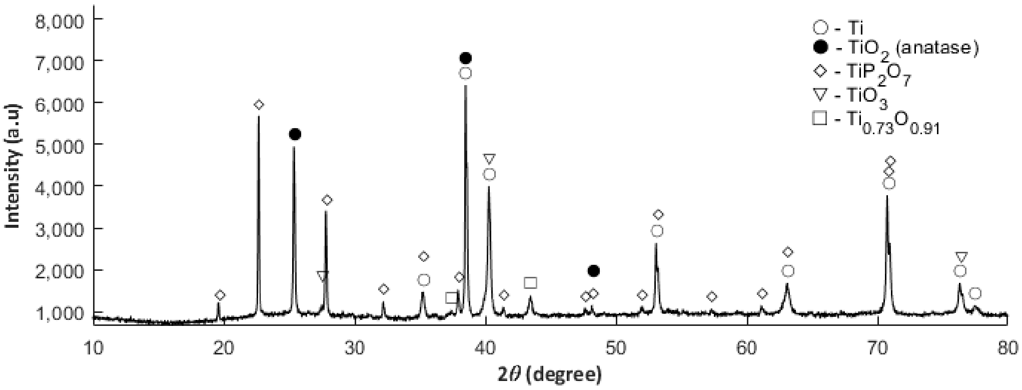

Figure S17. XRD diffractogram obtained for the PEO coating fabricated in the electrolyte consisting of orthophosphoric acid and copper(II) nitrate(V) trihydrate at concentration 10 g/dm³ with a voltage of 550 V.

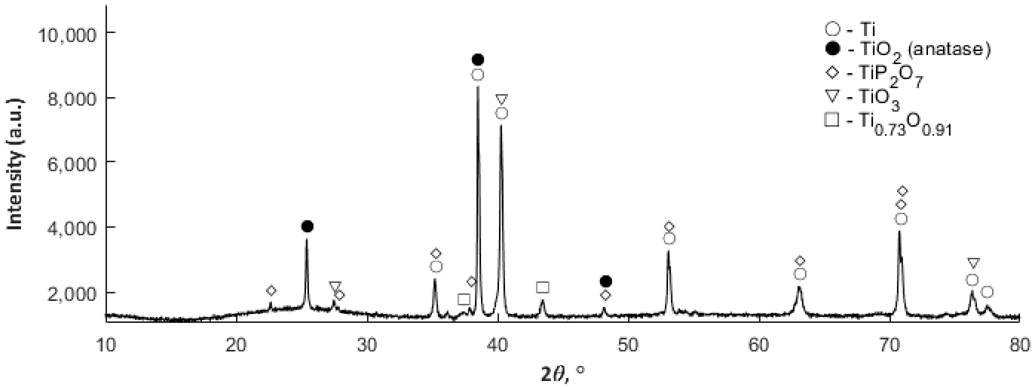

Figure S18. XRD diffractogram obtained for the PEO coating fabricated in the electrolyte consisting of orthophosphoric acid and copper(II) nitrate(V) trihydrate at concentration 50 g/dm^3 and voltage of 550 V.

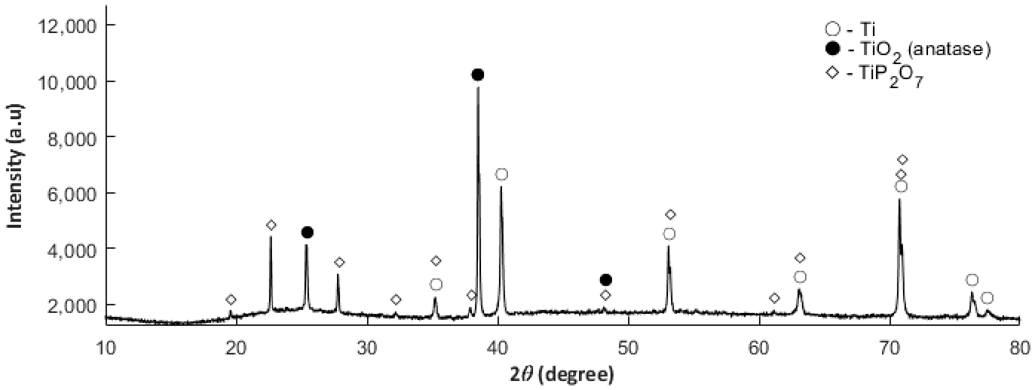

Figure S19. XRD diffractogram obtained for the PEO coating fabricated in the electrolyte consisting of orthophosphoric acid and copper(II) nitrate(V) trihydrate at concentration 200 g/dm^3 and voltage of 550 V.

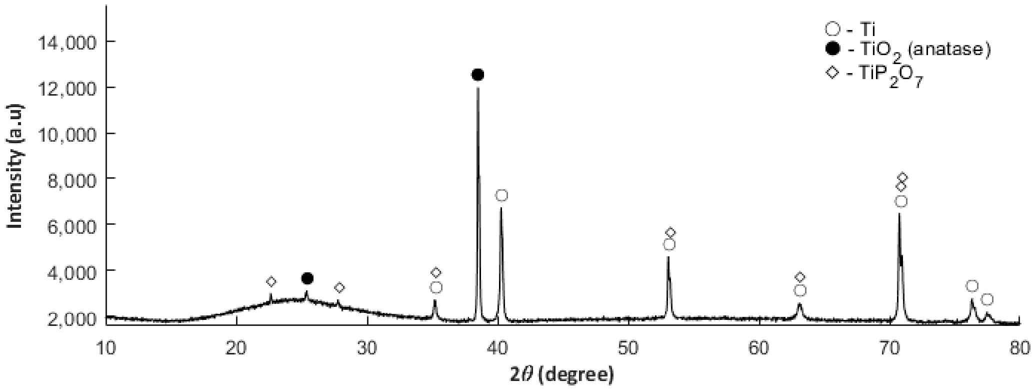

Figure S20. XRD diffractogram obtained for the PEO coating fabricated in the electrolyte consisting of orthophosphoric acid and copper(II) nitrate(V) trihydrate at concentration 350 g/dm^3 and voltage of 550 V.

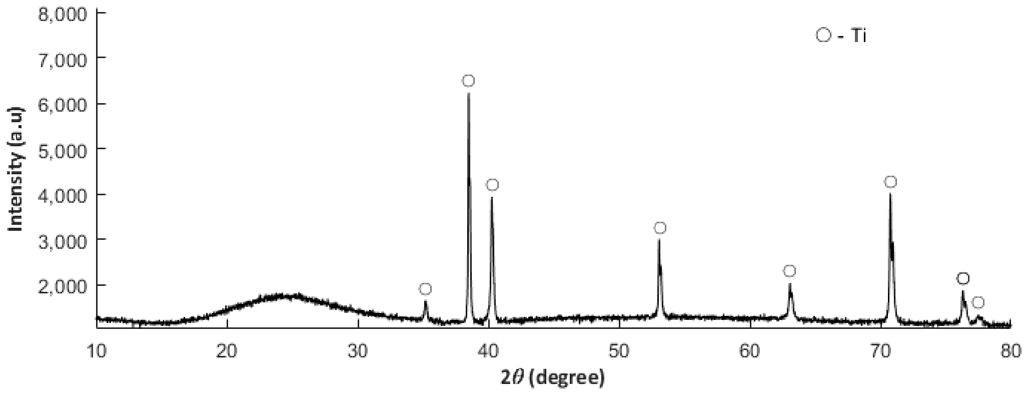

Figure S21. XRD diffractogram obtained for the PEO coating fabricated in the electrolyte consisting of orthophosphoric acid and copper(II) nitrate(V) trihydrate at concentration 500 g/dm^3 and voltage of 550 V.

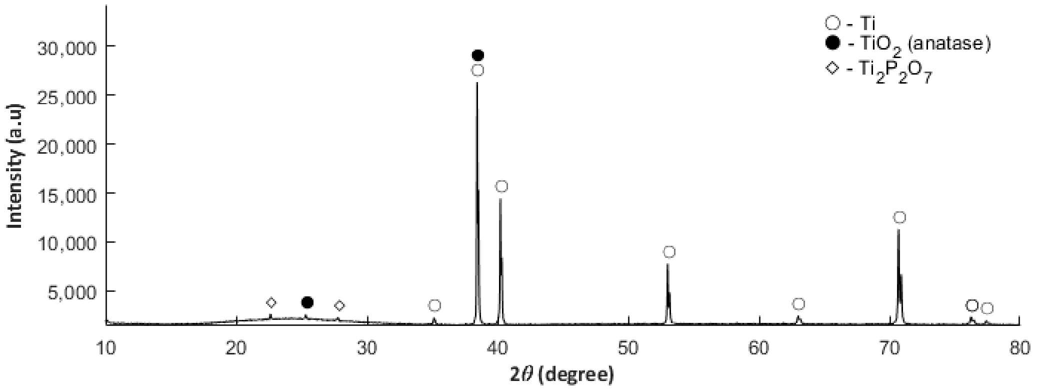

Figure S22. XRD diffractogram obtained for the PEO coating fabricated in the electrolyte consisting of orthophosphoric acid and copper(II) nitrate(V) trihydrate at concentration 500 g/dm^3 and voltage of 450 V.

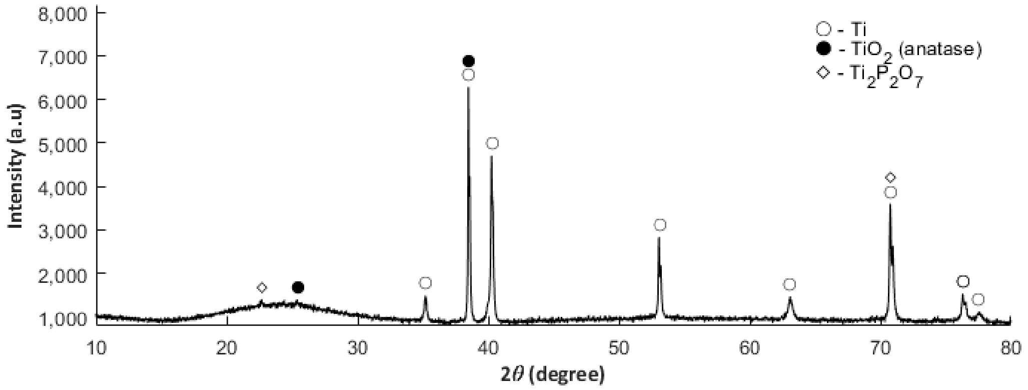

Figure S23. XRD diffractogram obtained for the PEO coating fabricated in the electrolyte consisting of orthophosphoric acid and copper(II) nitrate(V) trihydrate at concentration 500 g/dm^3 and voltage of 650 V.

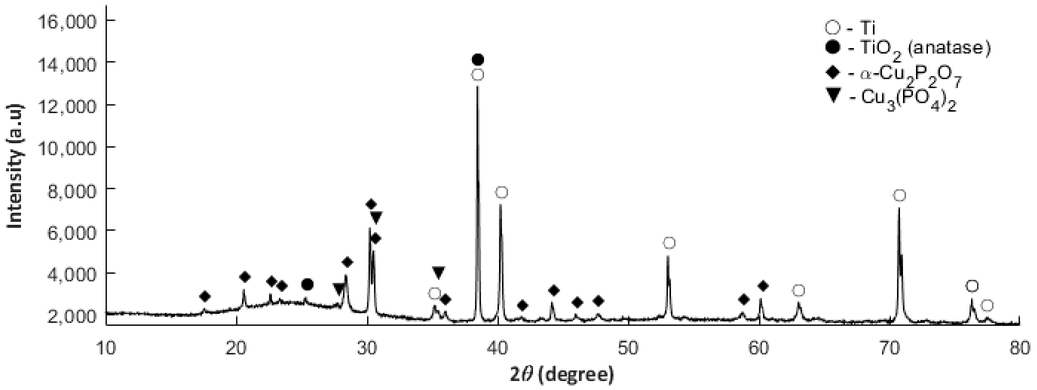

Figure S24. XRD diffractogram obtained for the PEO coating fabricated in the electrolyte consisting orthophosphoric acid and the copper(II) nitrate(V) trihydrate of concentration 650 g/dm^3 and voltage of 550 V.

© 2020 by the authors. Submitted for possible open access publication under the terms and conditions of the Creative Commons Attribution (CC BY) license (http://creativecommons.org/licenses/by/4.0/).
